# Norepinephrine induces anoikis resistance in high-grade serous ovarian cancer precursor cells

**DOI:** 10.1172/jci.insight.170961

**Published:** 2024-01-25

**Authors:** Hunter D. Reavis, Stefan M. Gysler, Grace B. McKenney, Matthew Knarr, Hannah J. Lusk, Priyanka Rawat, Hannah S. Rendulich, Marilyn A. Mitchell, Dara S. Berger, Jamie S. Moon, Suyeon Ryu, Monica Mainigi, Marcin P. Iwanicki, Dave S. Hoon, Laura M. Sanchez, Ronny Drapkin

**Affiliations:** 1 Penn Ovarian Cancer Research Center, Department of Obstetrics and Gynecology;; 2 Cell and Molecular Biology Graduate Group; and; 3 Department of Cancer Biology, University of Pennsylvania Perelman School of Medicine, Philadelphia, Pennsylvania, USA.; 4 Department of Chemistry and Biochemistry, University of California, Santa Cruz, Santa Cruz, California, USA.; 5 Division of Reproductive Endocrinology and Infertility, Department of Obstetrics and Gynecology, University of Pennsylvania Perelman School of Medicine, Philadelphia, Pennsylvania, USA.; 6 Department of Translational Molecular Medicine and Sequencing Center, Saint John’s Cancer Institute, Providence Health Services, Santa Monica, California, USA.; 7 Departments of Bioengineering, Chemistry, and Chemical Biology and Biological Sciences, Stevens Institute of Technology, Hoboken, New Jersey, USA.; 8 Basser Center for BRCA, Abramson Cancer Center, University of Pennsylvania School of Medicine, Philadelphia, Pennsylvania, USA.

**Keywords:** Cell biology, Oncology, Cancer, Cell migration/adhesion, Obstetrics/gynecology

## Abstract

High-grade serous carcinoma (HGSC) is the most lethal gynecological malignancy in the United States. Late diagnosis and the emergence of chemoresistance have prompted studies into how the tumor microenvironment, and more recently tumor innervation, may be leveraged for HGSC prevention and interception. In addition to stess-induced sources, concentrations of the sympathetic neurotransmitter norepinephrine (NE) in the ovary increase during ovulation and after menopause. Importantly, NE exacerbates advanced HGSC progression. However, little is known about the role of NE in early disease pathogenesis. Here, we investigated the role of NE in instigating anchorage independence and micrometastasis of preneoplastic lesions from the fallopian tube epithelium (FTE) to the ovary, an essential step in HGSC onset. We found that in the presence of NE, FTE cell lines were able to survive in ultra-low-attachment (ULA) culture in a β-adrenergic receptor–dependent (β-AR–dependent) manner. Importantly, spheroid formation and cell viability conferred by treatment with physiological sources of NE were abrogated using the β-AR blocker propranolol. We have also identified that NE-mediated anoikis resistance may be attributable to downregulation of colony-stimulating factor 2. These findings provide mechanistic insight and identify targets that may be regulated by ovary-derived NE in early HGSC.

## Abstract 2

Norepinephrine exposure promotes a prometastatic phenotype in fallopian tube epithelial cells that potentiates micrometastasis of high grade serous carcinoma precursor lesions to the ovary.

## Graphical abstract

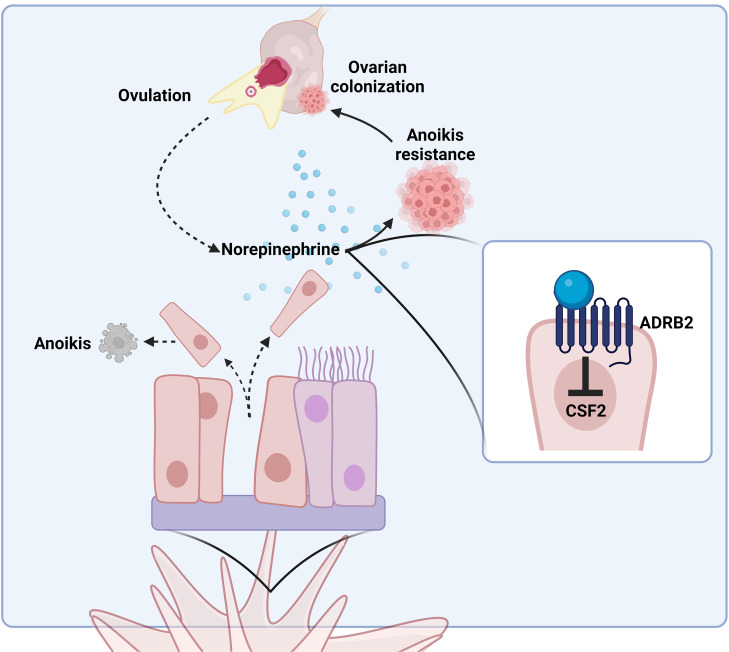

## Introduction

Ovarian cancer remains the deadliest of gynecological malignancies in the developed world, with approximately 19,680 new diagnoses and 12,740 deaths annually in the United States alone (1). While survival in early stages approaches 93%, the majority of ovarian cancers are diagnosed at or greater than stage III, leading to an overall 5-year survival rate of approximately 43% (2). Unfortunately, emerging early detection strategies to improve survival have thus far been unsuccessful (3, 4). Therefore, there is a pressing need to better understand the early events in ovarian cancer pathogenesis in order to develop screening and treatment modalities to reduce ovarian cancer deaths (5).

The most common type of epithelial ovarian cancer is high-grade serous carcinoma (HGSC), which accounts for 75% of ovarian cancer–related deaths (6–9). Since large tumors on the ovary are common at presentation, the ovarian surface epithelium was long considered to be the cell of origin of HGSC. However, over the past 20 years, strong evidence has emerged demonstrating that the secretory epithelial cells in the fimbriated end of the fallopian tube (FT) are the origin of most, if not all, HGSC (10–17). Initially identified in women undergoing risk-reducing salpingo-oophorectomies, serous tubal intraepithelial carcinomas (STICs) in the FT harbor the typical *TP53* mutations common to all HGSCs (11, 18–20). Multiple genomic studies have since identified a clonal relationship between concomitant STIC and HGSC lesions and that HGSC expression profiles more closely resemble fallopian tube epithelium (FTE) than ovarian surface epithelium (OSE) (15, 21). Moreover, STIC lesions can be found in women without a genetic predisposition to ovarian cancer at similar rates to women with *BRCA1/2* mutations, suggesting that sporadic cases of ovarian cancer likewise originate in the FT (12, 19, 22). Thus, FTE is now considered the predominant source of gynecologic HGSC, where precancerous lesions in the fimbriae ultimately metastasize to the ovary and become a dominant mass.

In order for aberrant FTE cells to give rise to a primary ovarian tumor, they must first be able to survive detachment from the fimbrial basement membrane. Normally, when healthy epithelial cells lose contact with their substrate, namely the extracellular matrix, they undergo a form of cell death known as anoikis (23–27). However, malignant cells can colonize distant sites throughout the body when they acquire mutations or other alterations that allow them to evade this particular form of cell death. This process of anchorage-independent survival is known as anoikis resistance, an essential step in transorgan migration during ovarian cancer development (28). Although this process is primarily characterized in the scope of metastasizing disease from a primary to secondary site, it is also a requisite of HGSC precursor cells preceding ovarian colonization.

One leading hypothesis of HGSC carcinogenesis originally described “incessant ovulation” as a stimulus for repetitive epithelial injury and repair, thereby leading to dysplasia and malignant transformation of the OSE (29). Although the site of origin for this disease has since been redefined, the association between ovulation and HGSC onset is still valid. Accordingly, factors that impact ovulation, such as parity, age of menarche, age of menopause, and oral contraceptive use, are correlated with cancer risk (30). This association may be explained by the repetitive exposure of the FTE to follicular fluid (FF) and other local factors during ovulation. To support this notion, it has been shown that in 2D FTE cell cultures, human FF induces inflammatory signaling, cell proliferation, and the characteristic mutant *TP53* accumulation seen in STIC (31–34), though the precise mechanisms remain unknown.

Over the past decade, evidence has accumulated to suggest that certain cancers, including ovarian cancer (35), can interact with the peripheral nervous system to potentiate tumor growth and metastasis (36). Norepinephrine (NE), one of the primary neurotransmitters of the sympathetic nervous system, signals to target cells via β-adrenergic receptors (β-ARs) and is elevated during ovulation, menopause, and polycystic ovarian syndrome (37–39). Epidemiologically, ovarian cancer patients with biobehavioral risk factors (stress, depression, low social support) have increased intratumor NE concentrations as well as increased activity of signaling pathways under β-adrenergic transcriptional control (40, 41). Moreover, some studies have identified a correlation between beta blocker use and improved ovarian cancer survival (42–44), although others have not (45–47). Interestingly, recent studies examining intercellular communication between murine ovary and FTE demonstrated that tumorigenic, but not normal, FTE cells can stimulate the release of NE from the ovary (48–50). This feed-forward loop enhances the migration of transformed FTE cells through Matrigel in vitro, providing preliminary evidence for a functional role of sympathetic stimulation in early HGSC. Several lines of evidence suggest that NE may be involved in advanced HGSC (35, 51–61), although much less is known in the context of early disease.

Therefore, the current study aimed to identify the effects of NE exposure on HGSC precursor cells. We postulated that ovary-derived NE exposure may promote a pro-metastatic phenotype that potentiates micrometastasis from the FT to the ovary, and we show for the first time to our knowledge that NE induces attachment-independent survival (anoikis resistance) in HGSC precursor cells through its canonical receptor, ADRβ2.

## Results

### NE induces spheroid formation and anoikis resistance in FTE cells.

**Figure 1:**
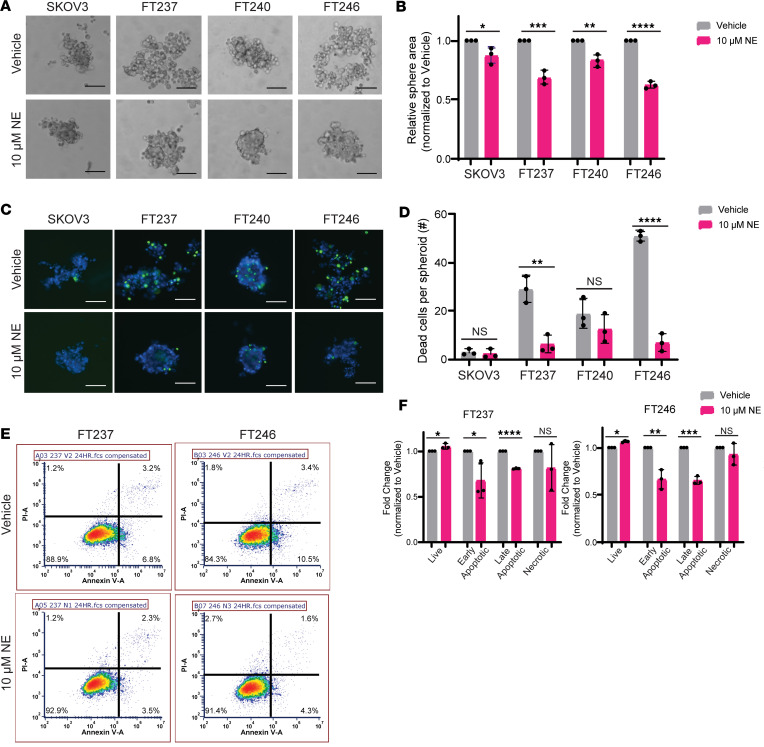
NE induces spheroid formation and anoikis resistance in FTE cells. (**A**) Bright-field images of the HGSC cell line SKOV3 as well as 3 immortalized human FTE cell lines (FT237, FT240, and FT246) cultured under ultra-low-attachment (ULA) conditions for 24 hours with vehicle or 10 μM NE. (**B**) Fold-change in average area for NE-treated spheres relative to vehicle-treated spheres as visualized in **A**. (**C**) ReadyProbe viability staining of cells cultured as in **A**. These images were taken at 10× original magnification and scale bars represent 100 µm. (**D**) Absolute quantification of dead cells (shown in green in **C**) per spheroid (shown in blue in **C**). (**E**) Representative flow cytometry plots for propidium iodide + annexin V staining, with quantification of cell populations relative to vehicle-treated cells in **F**, as cultured in **A**. Each experiment was conducted in technical triplicate for each biological replicate (*n* = 3). All statistical analyses for this figure were conducted with Student’s *t* tests. Data are represented as mean ± standard deviation. *P < 0.05, **P < 0.01, ***P < 0.001, ****P < 0.0001.

Spheroids are cellular structures formed when cells are placed into ultra-low-attachment (ULA) conditions and have the capacity for anchorage-independent survival and/or growth (62). This 3D model provides an in vitro system to study the morphology and survival of epithelial cells after detaching from their basement membrane, mimicking the functional events preceding in vivo metastasis. A panel of patient-derived FTE cell lines (FT189, FT190, FT194, FT237, FT240, FT246, FT282) were grown under ULA conditions in the presence or absence of NE. NE-induced spheroids appeared as tightly compacted groups of cells with few free-floating cells in the surrounding culture, similar to the morphology of the HGSC control cell line, SKOV3 (63). In comparison, vehicle-treated FTE cells remained in culture as a loose collection of singularly identifiable cells. More specifically, when compared with vehicle control, FT237, FT240, and FT246 exhibited significant compaction as measured by cross-sectional area, when exposed to 10 μM NE for 24 hours in culture (Figure 1, A and B). Four additional cell lines (FT189, FT190, FT194, and FT282) also formed tight spheroids upon treatment with 10 μM NE, although the effects were less pronounced (Supplemental Figure 1, A and B; supplemental material available online with this article; https://doi.org/10.1172/jci.insight.170961DS1). All of these FT cells had manipulation of *TP53* to enable immortalization. The most NE-sensitive cells (FT237, FT240, and FT246) were established via stable shRNA-mediated knockdown of *TP53*. The other FT lines were established with either SV40 Large T antigen expression (FT189, FT190, and FT194) or a TP53 mutation (FT282). It is possible that mutation or stabilization of p53 is associated with less sensitivity to NE treatment. It is also important to note that NE-mediated spheroid compaction occurred in a dose- and time-dependent manner, with the most distinct phenotypic differences observable upon 24 hours of treatment with 10 μM NE (Supplemental Figure 1, C–F).

To assess whether the observed compaction in response to NE was associated with cell death, cell staining was conducted using ReadyProbe fluorescent probes that selectively bind to DNA in dead cells (shown in green) and/or total cell spheroids (shown in blue) (Figure 1, C and D). As expected, cell death was readily detectable in the vehicle-treated FTE cells. Exposure to NE resulted in significantly fewer dead cells per spheroid in FT237 and FT246 when compared with vehicle control, resembling the anchorage-independent survival of SKOV3 cancer cells. While less pronounced, similar trends in NE-induced cell viability were observed in 5 additional FTE cell lines (FT189, FT190, FT194, FT240, and FT282) (Figure 1, C and D, and Supplemental Figure 2, A and B). To further substantiate these findings, propidium iodide (PI) and annexin V flow cytometry was performed to assess relative dead cell populations in response to NE in ULA culture (Figure 1E). In both FTE cell lines tested, NE exposure resulted in a significantly increased proportion of live cells and significantly fewer cells in the early and late apoptotic stages of cell death when compared with vehicle control (Figure 1F). NE exposure did not significantly impact the proportion of necrotic cells. Consistent with the sphere compaction data, anchorage-independent survival also occurred in a dose- and time-dependent manner (Supplemental Figure 2, C–F). Together, these results demonstrate that NE exposure promotes both spheroid formation and resistance to anoikis in immortalized FTE cells cultured under ULA conditions.

### Viable FTE precursor spheroids adhere to and displace OSE.

**Figure 2:**
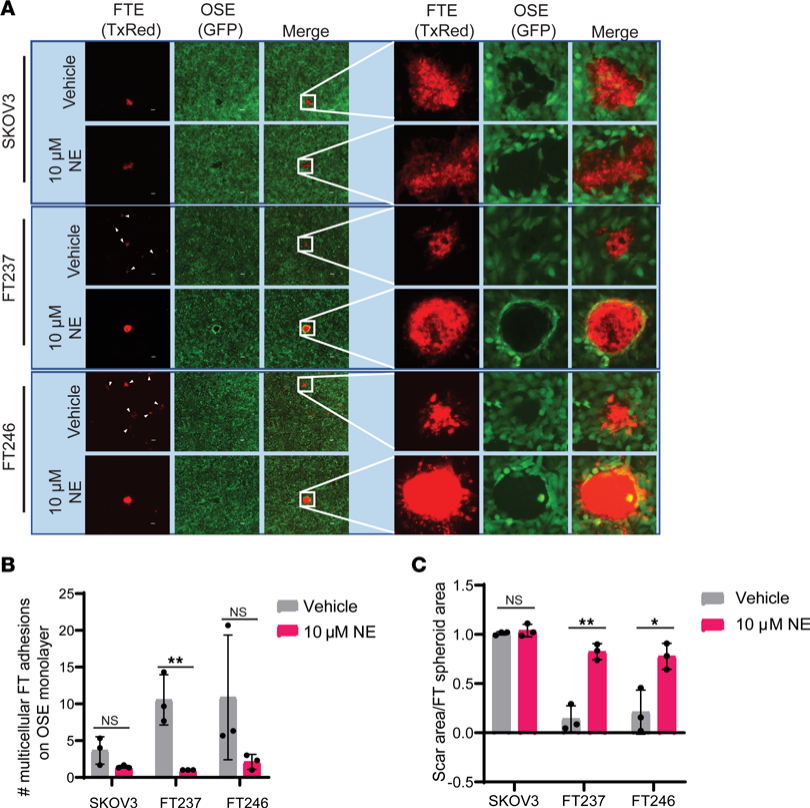
Viable FTE precursor spheroids adhere to and displace OSE. (**A**) Fluorescence images after 24 hours of coculture with 2D immortalized human OSE cells (HIO80-L2G, green) and CellTracker Red–stained FTE cells that were pretreated in ULA culture for 24 hours ± 10 μM NE. These images were taken at 4× original magnification, and insets are uniformly magnified regions of interest. (**B**) Quantification of the number of multicellular FTE cell adhesions (TxRed) on the GFP^+^ OSE monolayer. (**C**) Quantification of scar area in the OSE monolayer relative to the area of the adhered FTE cells. Each experiment was conducted in technical triplicate for each biological replicate (*n* = 3). All statistical analyses for this figure were conducted with Student’s *t* tests. Data are represented as mean ± standard deviation. *P < 0.05, **P < 0.01.

Primary HGSC tumors can arise from STICs or from *TP53*-mutant FTE cells that undergo precursor escape, whereby aberrant cells directly colonize the ovary without a detectable FTE lesion in situ (22, 64). In both of these scenarios, malignant FTE cells must acquire anchorage-independent survival characteristics in order to metastasize to the ovary. Given that NE promotes spheroid formation and anoikis resistance of HGSC precursors, we next asked whether NE-treated FTE spheres are capable of adhering to the OSE, the site of HGSC primary tumors. To address this possibility, FTE and SKOV3 HGSC control cells were transiently prestained with CellTracker Red and pretreated with or without NE in ULA culture to enable spheroid formation. After 24 hours in ULA culture, FTE and HGSC cells were seeded onto a monolayer of GFP^+^ OSE cells (HIO80-L2G) and incubated for an additional 24 hours. Nonadherent cells were then removed by consecutive washes prior to imaging. We observed that FTE cells from the vehicle-treated ULA culture were dispersed throughout the well upon transfer and settled on top of the OSE monolayer as small multicellular clusters (Figure 2, A and B). Strikingly, NE-treated spheroids maintained their dense multicellular structures upon transfer to the OSE monolayer and, once adhered, were able to displace existing OSE cells in 2D, forming a scar in the monolayer comparable to that of HGSC cells (Figure 2, A and C). These data show that NE not only confers ULA aggregation and anoikis resistance in FTE cells but also endows them with the ability to clear an ovarian epithelial monolayer, a trait necessary for primary HGSC tumor formation.

### NE induces anoikis resistance in an ADRβ2-dependent manner.

**Figure 3:**
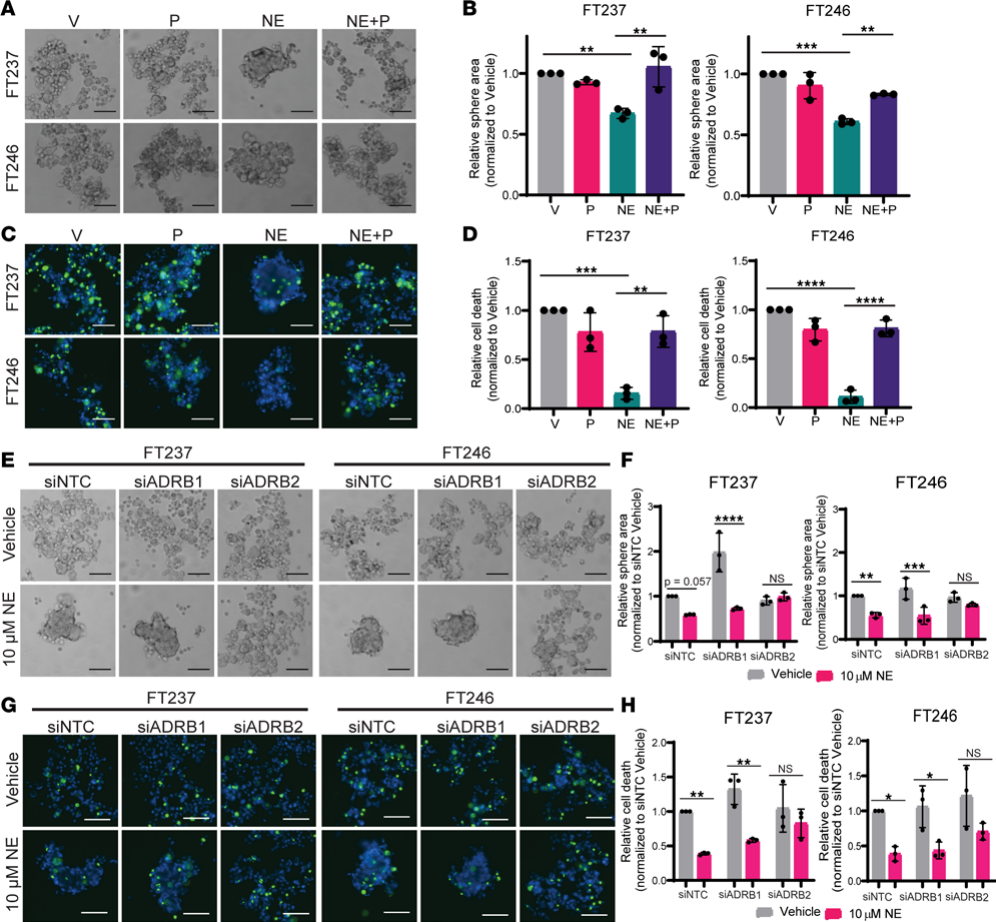
NE-induced anoikis resistance occurs in an ADRβ2-dependent manner. (**A** and **B**) Bright-field images and relative area quantification of FTE spheres cultured in ULA culture for 24 hours ± 10 μM NE (V, NE, respectively) and/or nonselective beta blocker, propranolol (NE+P, P, respectively). (**C** and **D**) Fluorescence images of ReadyProbe viability staining for spheroids (blue) and quantification of dead cells (green) cultured as in **A**. (**E**) Bright-field images for FTE cells cultured in ULA plates ± 10 μM NE after transfection with siNTC, siADRβ1, or siADRβ2. (**F**) Quantification of NE-treated spheroid area relative to vehicle-treated cells. (**G**) Fluorescence images of ReadyProbe viability staining for spheroids (blue) and quantification of dead cells (green) (**H**) in each siRNA condition. These images were all taken at 10× original magnification and scale bars represent 100 µm. Each experiment was conducted in technical triplicate for each biological replicate (*n* = 3). Statistical analyses were conducted with (**B** and **D**) 1-way ANOVA with Tukey’s correction for multiple comparisons or (**F** and **H**) 2-way ANOVA with Holm-Šídák correction for multiple comparisons. Data are represented as mean ± standard deviation. *P < 0.05, **P < 0.01, ***P < 0.001, ****P < 0.0001.

Intracellular NE-mediated signaling is initiated when the neurotransmitter binds to GPCRs collectively termed adrenergic receptors. In the scope of HGSC, independent studies indicate that the pro-tumorigenic effects of NE are mediated by β-ARs (51, 53–55, 57–59, 63), as opposed to α-adrenergic receptors (52, 56). We therefore postulated that β-ARs expressed by FTE cells may be responsible for NE-induced compaction and viability in anchorage-independent conditions. To test this, FT237 and FT246 cells were plated into ULA culture with NE or vehicle control, with or without the nonselective β-AR antagonist propranolol. In both cell lines, coculture with 10 μM propranolol resulted in significant attenuation of NE-induced spheroid compaction (Figure 3, A and B, and Supplemental Figure 3, A and B). Moreover, cotreatment with propranolol abrogated the NE-mediated decrease in dead cells per spheroid as measured using fluorescence viability cell staining (Figure 3, C and D, and Supplemental Figure 3, C and D). There was no observable effect of low-dose propranolol alone (≤ 10 μM) on spheroid formation or cell survival of vehicle-treated cells.

Since propranolol is a nonselective β-AR antagonist, we next assessed β-AR expression in FTE cells using reverse transcription quantitative PCR (RT-qPCR) to identify whether the observed phenotypes could be attributable to a particular receptor. All FTE and HGSC cell lines in the panel expressed similar amounts of *ADRβ1* and *ADRβ2*, albeit at lower levels than control tissues with known enrichments in β-AR expression (Supplemental Figure 4A). *ADRβ3*, the least common of the 3 β-AR subtypes, was not detected in most cell lines (data not shown).

To determine which receptor subtype is functionally responsible for mediating spheroid formation and anoikis resistance in this context, FT237 and FT246 cells were transfected with siRNAs targeting *ADRβ1* (siADRβ1), *ADRβ2* (siADRβ2), or nontargeted control (siNTC) (*n* = 4 siRNAs per pool) before ULA culture with or without NE (Supplemental Figure 4, B–D). As shown in Figure 3E, transfection with either siNTC or siADRβ1 did not impact NE-induced compaction, and thus, FTE cells produced robust spheres in both FT237 and FT246 (Figure 3F and Supplemental Figure 4E). However, knockdown of *ADRβ2* attenuated NE-induced spheroid formation. These results indicate that NE likely induces spheroid formation in an ADRβ2-dependent manner. Further, while siNTC- and siADRβ1-transfected cells still exhibited decreased cell death when treated with NE, there was a negligible difference in survival conferred by NE in siADRβ2-transfected cells (Figure 3, G and H, and Supplemental Figure 4F). Coupled with the propranolol data, these results ultimately indicate that NE-induced spheroid formation and anoikis resistance likely occurs in an ADR2-dependent manner. To verify the presence of ADRβ2 in human FT tissue, we conducted immunohistochemistry on 10 benign FT tissues from average-risk women and 10 benign FT tissues from high-risk patients harboring *BRCA1/2* mutations. In this cohort, we observed that the majority of samples exhibited medium to high ADRβ2 staining in the epithelial cells of the fimbria, regardless of BRCA status (Supplemental Figure 5). Together, these data support that ADRβ2 may indeed be active in early HGSC tumorigenesis.

### Propranolol abrogates morphological and survival changes imparted by NE-rich human FF.

Reproductive endocrinology literature suggests that FF, released from the granulosa cells within ovarian follicles during ovulation, contains NE (37, 38). Moreover, animal studies indicate that NE concentrations within the ovary increase with age (39). Taken together with the recent evidence of bidirectional communication between the ovary and HGSC precursor cells via NE (49), we postulated that NE within human FF may contribute to the development of HGSC through enhancement of anoikis resistance. To test this concept, we collected fresh FF from 17 patients undergoing oocyte retrievals for reproductive indications utilizing an IRB-approved protocol (Table 1).

**Figure 4:**
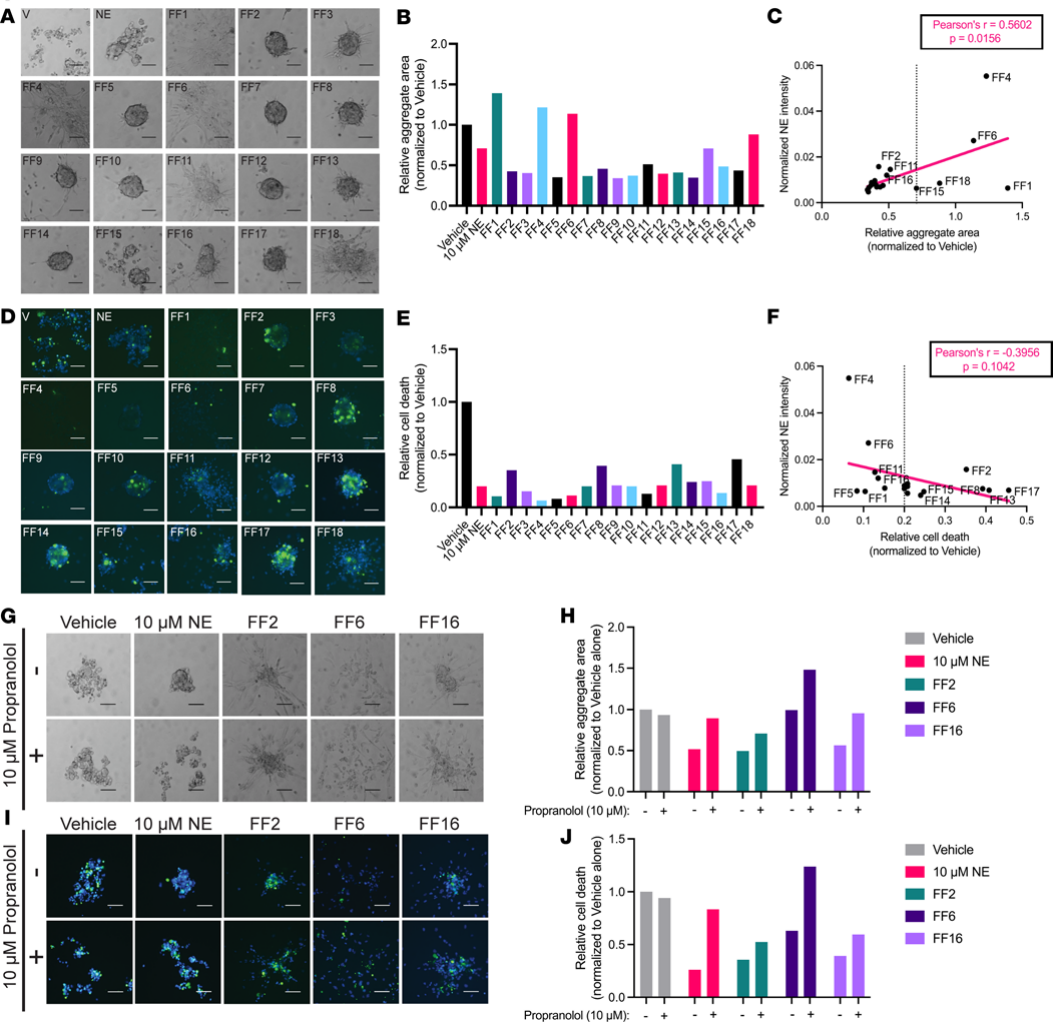
Propranolol abrogates morphological and survival changes imparted by NE-rich human FF. (**A** and **B**) Bright-field images and area measurements for FT246 cells treated for 24 hours in ULA culture with 18 different FF samples collected from 17 different patients (10% volume). (**C**) Linear regression for correlation between FT246 aggregate area and relative NE concentration quantified via mass spectrometry analysis. (**D**) Fluorescence images of total cells (blue) and dead cells (green), quantified in **E**, for each FT246 cell aggregate, cultured as in **A**. (**F**) Linear regression for correlation between the number of dead cells in FT246 aggregates and relative NE concentration per sample. (**G** and **H**) Bright-field images and area measurements for FT246 cells treated with vehicle, NE, FF2, FF6, or FF16 in the presence or absence of 10 μM propranolol. (**I** and **J**) Fluorescence images and dead cell quantification for cells as cultured in **G**. These images were all taken at 10× original magnification and scale bars represent 100 µm. Each experiment was conducted in technical triplicate for each biological sample (*n* = 1).

While treatment of FTE cells with FF led to heterogeneous changes in cell morphology under ULA conditions (Figure 4A and Supplemental Figure 6A), the majority of FF samples induced robust spheroid formation (Figure 4B and Supplemental Figure 6B). Surprisingly, samples containing higher levels of NE (FF4, -6, -16) were associated with larger, monolayer-like aggregate areas in FT246 cells (Figure 4C), while there was no observable correlation between NE concentration and aggregate area in FT237 (Supplemental Figure 6C). While FTE cell viability was also highly variable between FF samples, there was a universal decrease in the number of dead cells when compared to the vehicle-treated controls (Figure 4, D and E, and Supplemental Figure 6, D and E). In FT246, there was a slightly negative correlation between NE levels and cell death, indicating that samples with higher NE concentrations were largely associated with decreased cell death relative to samples with lower NE concentrations (Figure 4F). Consistent with the morphology results, there was no observable correlation between NE concentration and cell death in FT237 cells (Supplemental Figure 6F).

Based on the mass spectrometry analysis of relative NE concentrations (Table 1), 3 samples with high NE levels (FF2, FF6, FF16) were selected for further phenotypic testing with propranolol. While FF2- and FF16-treated cells did not exhibit significant differences, the addition of propranolol visibly altered the morphology of FF-treated FT246 cells, with increased dispersion of cells upon β-adrenergic inhibition (Figure 4, G and H). Most strikingly, in addition to changes in sphere compaction, propranolol treatment partially reversed cell survival imparted by FF6 in FT246 cells (Figure 4, I and J). While changes in propranolol-mediated sphere morphology were less robust in FF-treated FT237 cells (Supplemental Figure 6, G and H), cotreatment with propranolol generally increased the number of dead cells (Supplemental Figure 6, I and J). Together, these data suggest that FF, and endogenous NE, may confer β-AR–dependent changes in adhesion and survival in FTE cells, highlighting an important functional role for ovulation in pro-tumorigenic cell alterations.

### NE activates ADRβ-dependent transcriptional changes.

**Figure 5:**
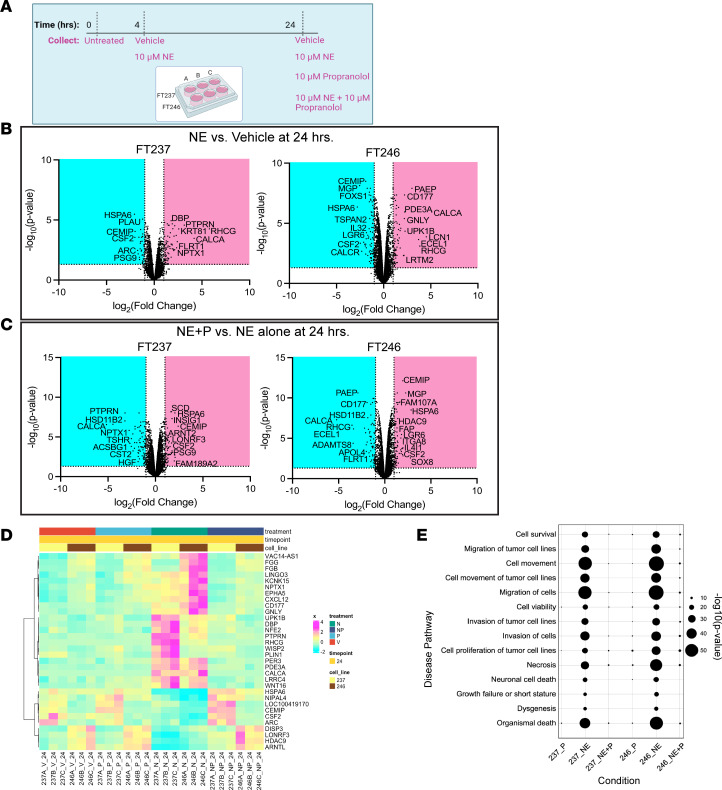
NE activates ADRβ-dependent transcriptional changes. (**A**) Experimental design for bulk RNA sequencing of FT237 and FT246 cells grown under ULA conditions for 4 and 24 hours, with or without 10 μM NE and/or 10 μM propranolol. (**B**) Volcano plots of differentially expressed transcripts upregulated (magenta) and downregulated (cyan) upon NE treatment relative to vehicle-treated cells at 24 hours. *CSF2*, colony-stimulating factor 2. (**C**) Volcano plots of differentially expressed transcripts upregulated (magenta) and downregulated (cyan) upon propranolol and NE treatment relative to NE treatment alone at 24 hours. (**D**) Heatmap of common transcripts between FT237 and FT246 cells whose NE-induced expression levels are abrogated upon cotreatment with propranolol at 24 hours; all transcript values are normalized to untreated 2D control cells. N, norepinephrine; NP, norepinephrine + propranolol; P, propranolol. (**E**) Ingenuity Pathway Analysis results for disease-related pathways enriched in NE-treated cells at the 24-hour time point.

In order to better characterize NE-induced functional pathways downstream of β-AR signaling in FTE cells grown under ULA conditions, bulk RNA sequencing was performed on FT237 and FT246 at 0, 4, and 24 hours in the presence or absence of NE and/or propranolol (Figure 5A). At 24 hours, treatment with 10 μM NE in ULA culture resulted in differential expression of 119 transcripts in FT237 cells (80 upregulated, 39 downregulated) and 169 transcripts in FT246 cells (75 upregulated, 94 downregulated) (Figure 5B). Relative to NE alone, the combination of NE and propranolol resulted in the differential expression of 167 transcripts in FT237 cells (76 upregulated, 91 downregulated) and 177 transcripts in FT246 (99 upregulated, 78 downregulated) (Figure 5C). Importantly, of the transcripts differentially regulated upon NE treatment relative to vehicle, 74% and 56% were reversed upon cotreatment with propranolol in FT237 and FT246, respectively.

Between FT237 and FT246, 22 transcripts were commonly upregulated in both cell lines upon NE treatment (relative to vehicle-treated cells) that were downregulated upon propranolol treatment (relative to NE alone). Also, 10 transcripts that were downregulated in both cell lines upon NE treatment were upregulated by cotreatment with propranolol. This indicates that a total of 32 transcripts were commonly altered in these FTE cells in an NE- and β-AR–dependent manner (Figure 5D and Table 2). Interestingly, many of these transcripts were also differentially regulated at the early 4-hour time point (Supplemental Figure 7), indicating a time-dependent element to NE/β-AR signaling FTE cells cultured in suspension. Ingenuity Pathway Analysis (QIAGEN) revealed that differentially expressed transcripts after 24-hour NE treatment in ULA culture were related to migration, invasion, and cell survival/death (Figure 5E).

### CSF2 knockdown recapitulates the NE-induced anoikis resistance phenotype.

**Figure 6:**
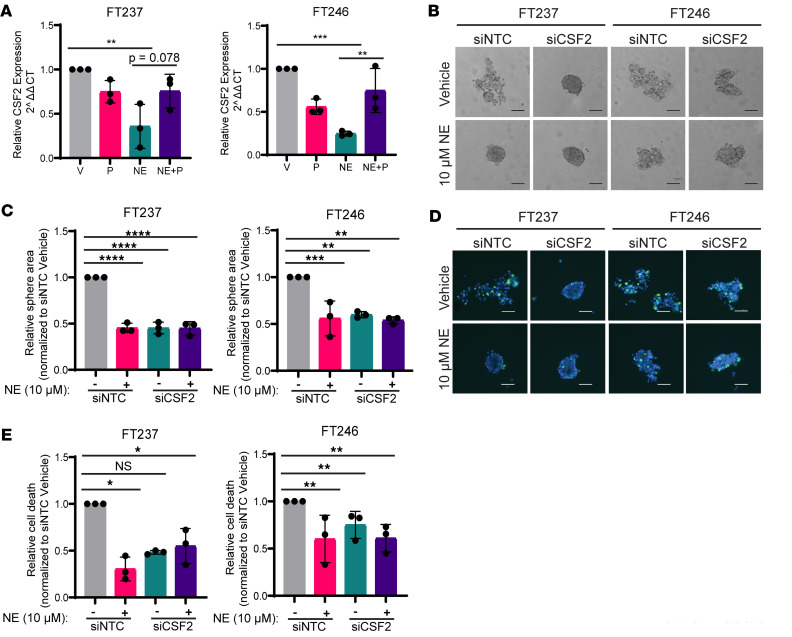
CSF2 knockdown recapitulates NE-induced anoikis resistance phenotype. (**A**) RT-qPCR validation of *CSF2* expression in FT237 and FT246 upon 24-hour treatment with vehicle, 10 μM propranolol, and/or 10 μM NE under ULA conditions. (**B**) Representative bright-field images of respective siNTC/siCSF2-transfected FTE cell lines cultured for 24 hours under ULA conditions ± 10 μM NE, quantified in **C**. (**D**) Representative fluorescence images of total cells (blue) and dead cells (green) in siNTC/siCSF2-transfected cells cultured as in **B**, quantified in **E**. These images were all taken at 10× original magnification and scale bars represent 100 µm. Each experiment was conducted in technical triplicate for each biological replicate (*n* = 3). All statistical analyses for this figure were conducted with 1-way ANOVA with either (**A**) Tukey’s or (**C** and **E**) Dunnett’s corrections for multiple comparisons. Data are represented as mean ± standard deviation. *P < 0.05, **P < 0.01, ***P < 0.001, ****P < 0.0001.

When comparing the most significantly differentially regulated transcripts in both cell lines (Figure 5, B and C, and Supplemental Figure 7A), *CSF2* stood out as being markedly downregulated by NE at both 4 and 24 hours, with rescued expression levels upon propranolol treatment in the 24-hour analysis. Validation of the bulk RNA-sequencing data indicated that NE- and propranolol-mediated changes in *CSF2*, among other hits, were reproducible via RT-qPCR (Figure 6A and Supplemental Figure 8). Interestingly, it did not appear that baseline *CSF2* expression levels in FTE cell lines were associated with NE-mediated anoikis resistance phenotypes (Supplemental Figure 9A). To investigate whether *CSF2* downregulation may be functionally responsible for NE-mediated sphere formation and ULA survival, FT237 and FT246 were transfected with a pool of siRNAs targeting *CSF2* or NTCs (*n* = 4 siRNAs per pool) (Supplemental Figure 9, B–G). Strikingly, cells with *CSF2* knockdown formed tight spheres in the absence of NE (Figure 6, B and C). Further, NE treatment did not significantly alter sphere formation or cell viability in siCSF2 cells (Figure 6, D and E). These data imply that *CSF2* may mediate anoikis resistance in FTE cells downstream of NE signaling, and further investigations are underway to define the mechanism by which this occurs.

## Discussion

In light of new evidence that peripheral nerves can initiate and potentiate tumor growth through delivery of neurotransmitters, such as NE (36, 41, 65, 66), we considered whether NE may play a role in the progression of HGSC from the FT. We utilized immortalized human FTE cell lines with reduced *TP53* expression (shTP53) (67, 68), characteristic of HGSC precursor lesions (13), and exposed them to a physiologically relevant dose of NE (69) that is also commonly used in relevant experimental reports (54, 70). Overall, we demonstrate that NE enhances FTE cell spheroid formation and subsequent viability in ULA culture conditions, enabling adhesion of these aggregates to the OSE. Functional siRNA knockdown indicates that this phenotype is primarily mediated by ADRβ2. In addition, we were able to demonstrate that NE-rich human FF confers changes in sphere morphology and survival that were partially reversible upon cotreatment with the β-AR antagonist propranolol. We also show that NE alters transcriptional cell migration, invasion, and viability pathways in HGSC precursors and that *CSF2* may be a regulator of anoikis resistance in FTE cells.

In HGSC, spheroids isolated from patient ascites samples can efficiently clear mesothelial cell layers in vitro, implying that spheroids likely seed peritoneal metastases (71–73). The current study demonstrates that NE may promote early precursor spheroid metastasis, as it enhances the ability of FTE cells to form viable spheroids that can adhere to and remodel the OSE. That NE can impart these cells with the capacity to survive in the absence of a basement membrane and remodel distant tissue highlights an important role of this neurotransmitter in regulating early disease onset. Anoikis resistance is a critical feature of HGSC that contributes significantly to tumor aggressiveness (28) and resistance to standard-of-care chemotherapeutic agents (74). This further emphasizes that understanding relevant mediators of anoikis resistance, especially at early stages, is crucial in HGSC progression.

In addition to correlative clinical studies (42, 43), several in vitro and in vivo experiments have suggested a role for β-AR inhibition in mitigating advanced HGSC progression (52–59, 63). Importantly, biobehavioral studies in mice have shown that when HGSC growth and metastasis are exacerbated by restraint stress, propranolol treatment efficiently reduces stress-mediated tumorigenesis (51). While these data suggest that propranolol administration may be beneficial for patients with HGSC after diagnosis, our data support that propranolol may have early interventional potential. With better understanding of the role of the neural milieu in tumor onset and progression, repurposing existing neurotransmitter-related drugs becomes a promising new therapeutic avenue in cancer prevention and/or treatment.

Little is known about the role of NE or the autonomic nervous system as it relates to normal ovarian function, despite histologic studies demonstrating rich sympathetic and parasympathetic innervation in the mammalian ovary (75). However, noradrenergic innervation of the ovary appears to influence ovarian steroidogenesis and follicle development and may play a causal role in the development of polycystic ovary syndrome (76–78). Most research into the effects of sympathetic innervation on cancer progression has thus far been approached from a psychosocial standpoint (40, 41, 51, 53, 55, 56, 58, 63). For the first time to our knowledge, we have connected ovulation-associated NE with HGSC. FF, or the fluid secreted by the granulosa cells of the ovary, accompanies the oocyte when it is released from the follicle during ovulation. It has been previously established that FF induces pro-tumorigenic changes in FTE cells (31) and that FF can promote anoikis resistance in transformed FTE and HGSC cells (34), but the role of NE in instigating these phenotypes in FTE cells has never been characterized to our knowledge. In our study, the abrogation of FF-mediated ULA cell survival upon β-AR inhibition with propranolol indicates that this source of NE may promote FTE cell anoikis resistance and ultimately give rise to HGSC. However, it is important to acknowledge that there was interpatient heterogeneity in NE concentrations as well as phenotypic effects of each FF sample on FTE cells. This is likely attributable to the variety of other biological components in FF and the factors contributing to their variance (79); because these samples were collected from patients undergoing hormonally stimulated oocyte retrieval, the FF samples used may not fully represent FF released during physiologic ovulation. With the current sample size (*n* = 18), we did not observe any association between race or ethnicity with respect to NE levels. It will be important to evaluate this in more diverse patient populations. Interestingly, there was a modest correlation between relative NE concentrations and progesterone levels, as well as the number of oocytes retrieved in each patient (Table 1). Whether or not these findings are functionally relevant remains to be determined. Recent studies suggest that in addition to ovulation, ovarian NE increases with age and reproductive senescence (39, 80), raising the possibility of increased NE in the FF of perimenopausal women. That ovary-derived NE concentration increases with age (80) may provide an explanation for the predicted latency between the development of an STIC and symptomatic disease in sporadic ovarian cancer (14).

There is a growing body of literature detailing the transcriptional changes of acute and chronic NE treatment on adherent FTE cells in vitro (70, 81). Interestingly, we found little overlap between these analyses and the results of our bulk RNA sequencing, indicating that there may be a distinct transcriptional profile for HGSC precursor cells exposed to NE that is necessary for, or driven by, ULA culture. Of interest, our analysis indicated that NE treatment led to a decrease in *CSF2* RNA expression levels. With respect to anoikis resistance, a recent study in small-cell lung cancer reported that adherent cells express significantly more CSF2 than subclones of the same cell line that can survive in suspension (82). Interestingly, titration with human recombinant CSF2 (0.1 ng/mL to 1 μg/mL) was unable to rescue anoikis in NE-treated spheroids (data not shown). It is therefore possible that CSF2 has an intracellular role, independent of the extracellular CSF2 receptor, although further investigation is required to support this. While the exact mechanism remains to be determined, our functional studies with *CSF2*-knockdown FTE cells support an active role for this gene in enabling spheroid compaction and survival in the absence of an adhesive substrate.

Together, the data presented herein support the hypothesis that FF-derived NE potentiates the progression of HGSC from FTE precursor lesions by enhancing anchorage-independent cell survival that is a prerequisite for coelomic spread and disseminated disease. These findings lend credence to the hypothesis of incessant ovulation as an inciting event of HGSC and realigns the classic risk factor (29) according to current perspectives on the tubal origins of this deadly disease. Further research is warranted into the effects of ovulation-associated NE on HGSC carcinogenesis and its potential as a target for preventative and/or therapeutic intervention.

## Methods

### Sex as a biological variable.

All biological samples, tissues, and cell lines in this manuscript were obtained from assigned females at birth. This is because HGSC is a disease that only occurs in assigned females at birth.

### Reagents and materials.

NE was purchased from MilliporeSigma (catalog A7257) and Cayman Chemical Company (catalog 16673). Propranolol hydrochloride (catalog P0844) was purchased from MilliporeSigma. Compounds were reconstituted per manufacturer’s protocol.

### Tissue culture.

All FT cell lines (FT189, FT190, FT194, FT237, FT240, FT246, FT282) were established in our lab and cultured in DMEM:F12 (Gibco) + 1% Penicillin/Streptomycin (Gibco) + 2% Ultra-Ser G (Sartorius), as previously described (67, 68). SKOV3 cells were purchased from ATCC and were cultured in McCoy’s Media (Gibco) + 1% Penicillin/Streptomycin (Gibco) + 10% fetal bovine serum. All other HGSC cell lines (CaOV3, EFO27, Kuramochi, OVCAR4, OVCAR8) were cultured in RPMI (Gibco) + 1% Penicillin/Streptomycin (Gibco) + 10% fetal bovine serum. CaOV3 cells were obtained from ATCC. EFO27 cells were a gift from Gottfried Konecny (University of California, Los Angeles, Los Angeles, California, USA). Kuramochi cells were obtained from the Japanese Collection of Research Bioresources Cell Bank (Japan). OVCAR4 cells were a gift from Tom Hamilton (Fox Chase Cancer Center, Philadelphia, Pennsylvania, USA). OVCAR8 cells were a gift from William Hahn (Dana Farber Cancer Institute, Boston, Massachusetts, USA). OSE cells (HIO80) were a gift from Andrew Godwin at the University of Kansas Medical Center (Kansas City, Kansas, USA) and were cultured in a 1:1 solution of MCDB105 (Cell Applications) and M199 (Gibco) cell culture media + 1% Penicillin/Streptomycin (Gibco) + 10% fetal bovine serum. Cells were maintained in a 37°C incubator with 5% CO_2_. All cell lines were routinely evaluated and tested negative for mycoplasma.

### Sphere formation assay.

Prior to cell seeding, wells of a 96-well, ULA plate (Corning) were pretreated with vehicle, 10 μM NE, and/or 10 μM propranolol unless otherwise noted. Cells grown in 2D (60%–80% confluence) were then trypsinized, resuspended, and counted. About 500 cells were plated in each well, and plates were incubated for 24 hours (unless otherwise noted) prior to bright-field imaging using a Nikon microscope at 10× original magnification. Bright-field images were analyzed by removing background and quantifying individual sphere area using ImageJ (NIH). Sphere areas were then normalized to the area of vehicle-treated cells. All experiments were conducted in biological and technical triplicate for each cell line unless otherwise noted. All scale bars represent 100 μm unless otherwise noted.

### Immunofluorescence.

For the fluorescence viability staining, spheres were stained with ReadyProbe reagents (Invitrogen) per manufacturer’s protocol and imaged using DAPI (total cells) and FITC (dead cells) channels of a Nikon microscope at 10× original magnification. The overall number of dead cells was counted for each spheroid. Relative cell death was determined by normalizing dead cell counts to those of the vehicle-treated cells. All experiments were conducted in biological and technical triplicate for each cell line unless otherwise noted. All scale bars represent 100 μm unless otherwise noted.

### Flow cytometry.

Prior to cell seeding, wells of a 6-well, ULA plate were pretreated with vehicle or 10 μM NE. A minimum of 250,000 cells/well were plated in each well. After a 24-hour incubation, suspended cells were collected, trypsinized, neutralized, and washed once with 1× PBS (Gibco) prior to staining with the Dead Cell Apoptosis Kit (Annexin V – Alexa Fluor 488 + PI, Thermo Fisher Scientific) per manufacturer’s protocol. Flow cytometry was conducted on the Accuri C6 at the Flow Cytometry Core Laboratory at the Children’s Hospital of Philadelphia. Cells were first gated to eliminate debris and doublets. Fluorescent cells were then further gated using unstained controls, and data were analyzed using FCS Express 7 (De Novo Software). Relative changes in cell populations were compared with vehicle-treated cells.

### OSE clearance assay.

HIO80 OSE cells were stably infected with the UBC-GFP-T2A-Luciferase lentivector (GFP) (System Biosciences). Following infection, cells were sorted using FACSJazz (BD), and the top 10% of GFP-positive cells were collected. FT237, FT246, and SKOV3 cells were transiently stained using CellTracker Red CMPTX dye (Invitrogen) per manufacturer’s protocol. On the day of seeding, 500 FTE/HGSC cells were plated into ULA without/with vehicle or 10 μM NE, and 50,000 GFP^+^ HIO80 cells were plated into a flat-bottom, 2D, 96-well culture plate. At 24 hours after seeding, media and FTE cells from ULA culture plates were added to the 2D OSE cultures. After a further 24-hour incubation, nonadherent cells were removed through 3 PBS (1×, Gibco) washes prior to imaging. Cocultures were then imaged using FITC and TxRed filters of a Nikon microscope at 4× original magnification. The overall number of multicellular adhesions was counted manually. Scar areas were calculated using ImageJ and were normalized to the size of the FTE cell(s) in the given region of interest.

### RT-qPCR.

RNA was isolated from all samples in biological triplicate using the Norgen RNA isolation kit. RNA quality and concentration were assessed by NanoDrop prior to High Capacity cDNA Reverse Transcription Kit (Applied Biosystems, Thermo Fisher Scientific). RT-qPCR was conducted in technical triplicate using PowerUp SYBR Green Master Mix (Thermo Fisher Scientific) on a QuantStudio Flex 6 system. RT-qPCR primers (Supplemental Table 1) were designed using the Massachusetts General Hospital/Harvard PrimerBank database, Primer3, and National Center for Biotechnology (NCBI) BLAST for specificity verification.

For ADRβ expression panels, control tissues (brain, heart, lung) were collected from 6- to 8-week-old female C57BL/6 mice (The Jackson Laboratory), flash-frozen, and homogenized using a mortar and pestle. All cell lines (FTE + HGSC) were cultured in 2D as described above and collected once cells had reached 60%–80% confluence. Fold change was calculated using the ΔΔCT method, first normalizing transcripts of interest to the housekeeping gene GAPDH, then to ADRβ expression levels in the mouse brain (positive control).

For relative RNA quantification between ULA samples, ΔΔCT was calculated by normalizing to GAPDH and the siNTC or vehicle conditions.

### RNA interference.

All siRNA gene-silencing experiments were conducted using the ON-TARGETplus human SMARTPool system from Horizon (siNTC: D-001810-10-05, siADRβ1: L-005425-00-0005, siADRβ2: L-005426-01-0005, siCSF2: L-011166-00-0005). Briefly, 50,000 cells were seeded into each well of a 6-well, flat-bottom plate in 2D and allowed to grow for 24 hours before transfection using Lipofectamine RNAiMAX (Invitrogen) per manufacturer’s protocol. Then, 24 hours later, medium was refreshed, and cells were incubated for another 24 hours prior to harvesting for downstream analyses. Following initial SMARTPool results, each siRNA within the pool was tested for relative knockdown efficiency using RT-qPCR (Supplemental Figure 4D and Supplemental Figure 9C).

### Western blotting.

Following transfection in 2D, cells were trypsinized, washed in 1× PBS (Gibco), pelleted, and resuspended in Pierce RIPA buffer (Thermo Fisher Scientific) complemented with Halt Phosphatase and Protease inhibitors (Thermo Fisher Scientific). Lysates were vortexed for 15 seconds per sample and incubated for 60 minutes on ice prior to Pierce BCA (Thermo Fisher Scientific) protein estimation per manufacturer’s protocol. Samples were diluted to equal concentrations in complemented RIPA buffer and 1× Laemmli SDS Reducing Buffer (Thermo Fisher Scientific). A total of 20 μg of each sample was loaded into respective wells of a 4%–15% Tris-Glycine gel (Bio-Rad). After running at 120 V until appropriate protein separation was observed, the gel was transferred onto PVDF (Bio-Rad) using a Bio-Rad Trans-blot Turbo semidry transfer system. Membranes were then blocked in 5% nonfat dry milk (Lab Scientific) prior to overnight 4°C incubation with GAPDH (Cell Signaling Technology: 97166S) or ADRβ2 (MilliporeSigma: HPA003431) antibodies diluted at 1:1,000 in 5% milk. After 3 TBST (1×, Bio-Rad) washes, membranes were incubated with HRP-conjugated secondary antibodies (Cell Signaling Technology) diluted at 1:1,000 in 5% milk. Following an additional three 1× TBST washes, SuperSignal ECL (Thermo Fisher Scientific) was applied to the membranes, and they were developed using a Bio-Rad ChemiDoc MP system.

### Immunohistochemistry.

For this study, 20 formalin-fixed, paraffin-embedded human FT samples were sectioned and stained by immunohistochemistry using a rabbit monoclonal ADRβ2 antibody from NSJ Bioreagents (RQ4480) at a concentration of 1:250. Staining was scored as negative, 0; low, 1; moderate, 2; or high, 3.

### FF acquisition.

Human FF samples were collected from 17 women undergoing ultrasound-guided oocyte retrieval for assisted reproductive technologies. Samples FF7 and FF8 were from the same patient, giving us a total of 18 samples. Sample allocation for this study was conducted in accordance with the University of Pennsylvania IRB. Upon extraction, FF was placed on ice for transport and spun down at 2,000*g* for 10 minutes in order to remove red blood cell debris. The supernatant was then aliquoted and samples were stored at –80°C. For experimental use, samples were diluted to 100 μL/mL (determined by titration based on ref. 83) in FT cell culture media (described above).

### Mass spectrometry for NE quantification.

Stock solutions of heavy standards were prepared at 100 μM concentrations in Milli-Q water (MilliporeSigma). FF samples (100 μL each) were spiked with 50 μM norepinephrine-D6 (MilliporeSigma). The spiked FF samples were subjected to solid-phase extraction using a Bond Elut PBA column (Agilent). The column was primed using 3 mL 0.1% ammonium formate (MilliporeSigma) at pH 3, followed by 3 mL 60:40 MeOH:ACN and 3 mL 0.1% ammonium formate at pH 10. One milliliter of 0.1% ammonium formate at pH 10 was added before loading on the PBA column. After loading, samples were washed with 3 mL 0.1% ammonium formate at pH 10 followed by 3 mL MeOH:ACN. HPLC-grade methanol was purchased from MilliporeSigma. HPLC-grade formic acid was purchased from Thermo Fisher Scientific. Catecholamines were eluted using 3 mL 0.1% ammonium formate at pH 3, and samples were dried in vacuo.

Reverse-phase LC was performed on an Elute ultra-high-performance liquid chromatography system (Bruker Daltonics) using a Phenomonex Kinetex biphenyl C18 HPLC column (2.6 μm, 2.1 mm × 150 mm, 100 Å) with a sample injection volume of 20 μL. The mobile phase consisted of A (H_2_O with 0.1% formic acid) and B (MeOH with 0.1% formic acid) with a flow rate of 0.3 mL/min. The gradient began with 1% B for 0.5 minute and was linearly increased to 5% B over 4 minutes. The column was washed using 90% B for 5 minutes and re-equilibrated using 1% B for 5 minutes. The temperature of the column oven was 40°C. Mass spectrometry spectra were collected using a timsTOF fleX mass spectrometer (Bruker Daltonics) in positive-ion mode with a mass range of 80–500 Da and a spectra rate of 4 Hz. Before analysis the instrument was calibrated using 0.5 mm sodium formate. The [M+H-H_2_O]^+^ adduct of norepinephrine at *m/z* 152.071 and the [M+H-H_2_O]^+^ adduct of norepinephrine-D6 at *m/z* 158.118 are the most abundant adducts; thus, these signals were used to calculate the normalized response for the relative quantification of NE.

### RNA sequencing.

FT237 and FT246 cells (>250,000 cells) were grown in biological triplicate in ULA plates for 0, 4, and/or 24 hours in the presence of vehicle, 10 μM NE, and/or 10 μM propranolol as previously described. After the allotted time in culture, cells were collected, and RNA was isolated using the Norgen Total RNA isolation kit. RNA quality was assessed using NanoDrop, and only samples with OD_260/280_ = 1.8–2.2 and RNA integrity number scores greater than 7 were used for sequencing. In collaboration with the Children’s Hospital of Philadelphia Center for Applied Genomics, samples were prepared using the TrueSeq Total RNA library with ribosomal depletion. Sequencing was then conducted using an S2 Flow cell with 3.3 billion to 4.1 billion read cluster capacity. Bulk RNA-sequencing data were analyzed in collaboration with the Translational Molecular Medicine and Sequencing Center at Saint John’s Cancer Institute. Significantly differentially expressed transcripts were determined at *P* < 0.05 and log_2_(fold-change) < –1 or > 1.

Ingenuity Pathway Analysis was conducted for each cell line, comparing expression profiles of transcripts in propranolol, NE, and the combination to the levels of the vehicle-treated cells.

### Statistics.

Each data point on bar graphs represents 1 replicate (biological or technical, as noted), and error bars represent the mean ± standard deviation. Student’s 2-tailed *t* tests were utilized to compare 2 independent groups. One-way ANOVA was used when comparing multiple groups with 1 variable. Dunnett’s correction for multiple comparisons was used when each group was compared with the control in 1-way ANOVAs. Tukey’s correction for multiple comparisons was used when each group was compared with every other group in 1-way ANOVAs. Two-way ANOVA was used when comparing multiple groups with multiple variables. Holm-Šídák test was used to compare vehicle- versus NE-treated conditions when multiple variables were present (siRNA, FF) in 2-way ANOVAs. *P* < 0.05 was considered statistically significant.

### Study approval.

Human FF samples were collected in accordance with the protocol 850118 titled “Effects of follicular fluid on human fallopian tube cells,” as approved by the University of Pennsylvania IRB on November 5, 2021. FT tissues were collected in accordance with protocol 702679 titled “Gynecologic oncology Bio-specimen repository,” as approved by the University of Pennsylvania IRB on July 14, 2020.

### Data availability statement.

Bulk RNA-sequencing data have been deposited and are accessible in the NCBI Gene Expression Omnibus database at accession GSE228234. Mass spectrometry results for FF NE levels are available via MassIVE data set MSV000091519. Supporting Data Values associated with all main and supplemental figures are available as an XLSX file.

## Author contributions

This project was conceived of and designed by HDR, SMG, and RD. Co–first authorship of HDR and SMG is reflected by their joint intellectual contributions and writing of this manuscript. Order of co–first authorship was determined by relative experimental/data contributions. HDR conducted experiments with general assistance from GBM and HSR and acquired/analyzed all subsequent data. MK provided reagents and technical assistance and helped with data interpretation for flow cytometry experiments. HJL and LMS conducted mass spectrometry analysis and aided in data interpretation of FF samples procured with the assistance of SMG, DSB, and MM. RNA-sequencing analysis was conducted by PR, JSM, SR, and DSH. MPI assisted with the experimental design and interpretation of data for the clearance assay. MAM and HSR were essential for reagent procurement.

## Supplementary Material

Supplemental data

Unedited blot and gel images

Supporting data values

## Figures and Tables

**Table 1:**
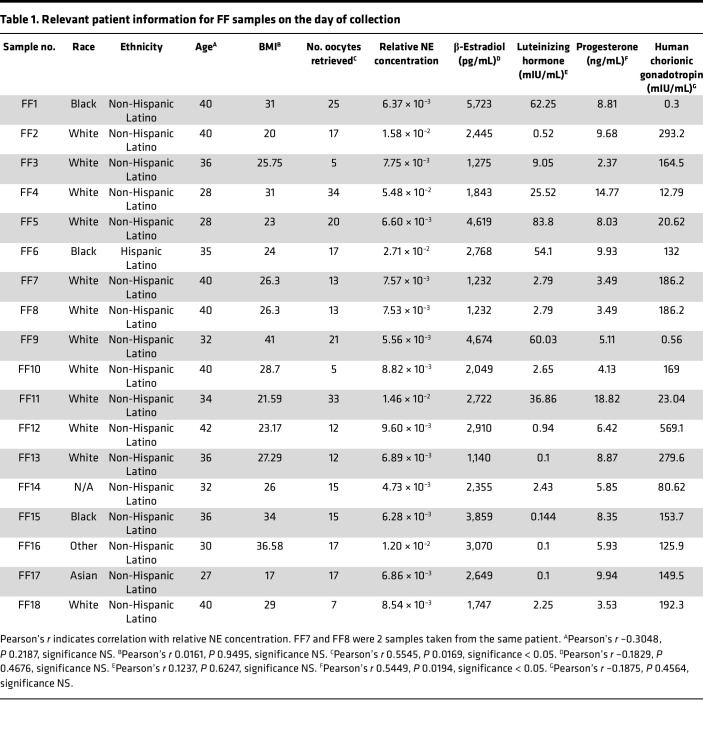
Relevant patient information for FF samples on the day of collection

**Table 2:**
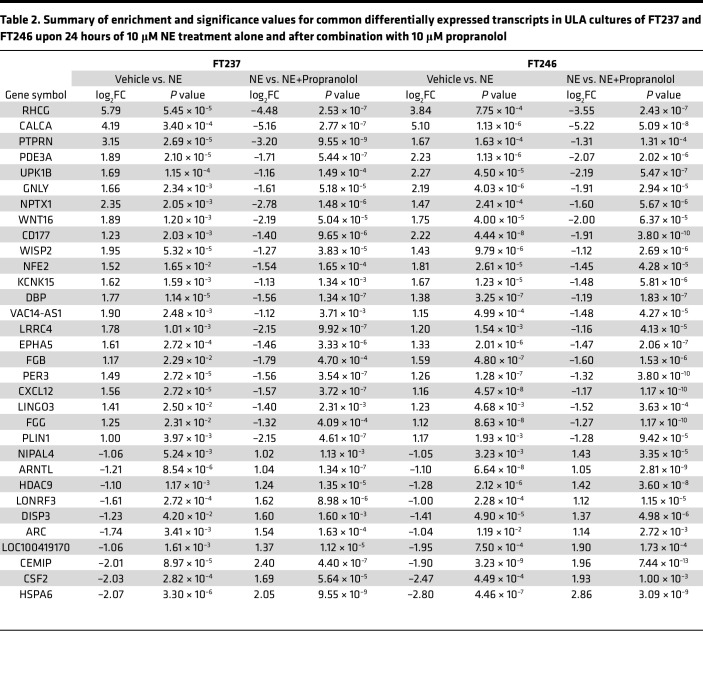
Summary of enrichment and significance values for common differentially expressed transcripts in ULA cultures of FT237 and FT246 upon 24 hours of 10 μM NE treatment alone and after combination with 10 μM propranolol

## Version 1 01/25/2024

In-Press Preview

## Version 2 03/08/2024

Electronic publication
