# Supplementary material for: Norepinephrine induces anoikis resistance in high-grade serous ovarian cancer precursor cells
Source: JCI Insight. 2024 Jan 25;9(5):e170961. doi: 10.1172/jci.insight.170961 (PMC10972597; doi:10.1172/jci.insight.170961)
Supplement: Supplemental data [file jciinsight-9-170961-s043.pdf]

Supplemental Figure 1

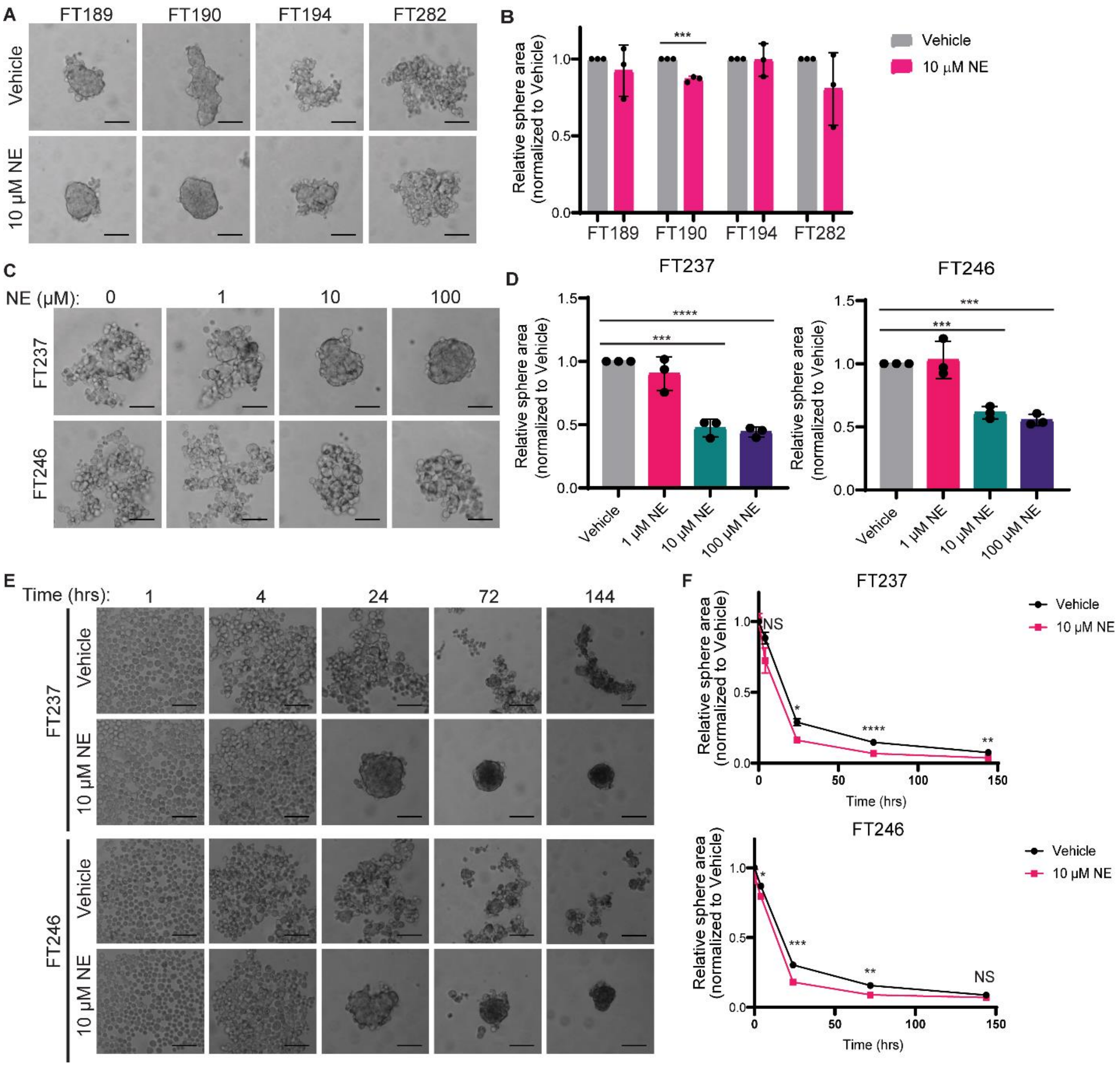

Supplemental Figure 1: Brightfield images and quantification of ultra-low attachment (ULA) spheroid areas for (**A,B**) 4 additional immortalized FTE cell lines (FT189, FT190, FT240, FT282) grown for 24 hrs -/+ 10μM NE, (**C,D**) FT237 and FT246 cells grown for 24 hrs in various concentrations of NE, (**E,F**) FT237 and FT246 cells grown -/+ 10μM NE at various timepoints. Statistical analyses were conducted via: (**B,F**) Student's t-tests, or (**D**) one-way ANOVA with Dunnett's correction for multiple comparisons. Data are represented as mean ± standard deviation.

Supplemental Figure 2

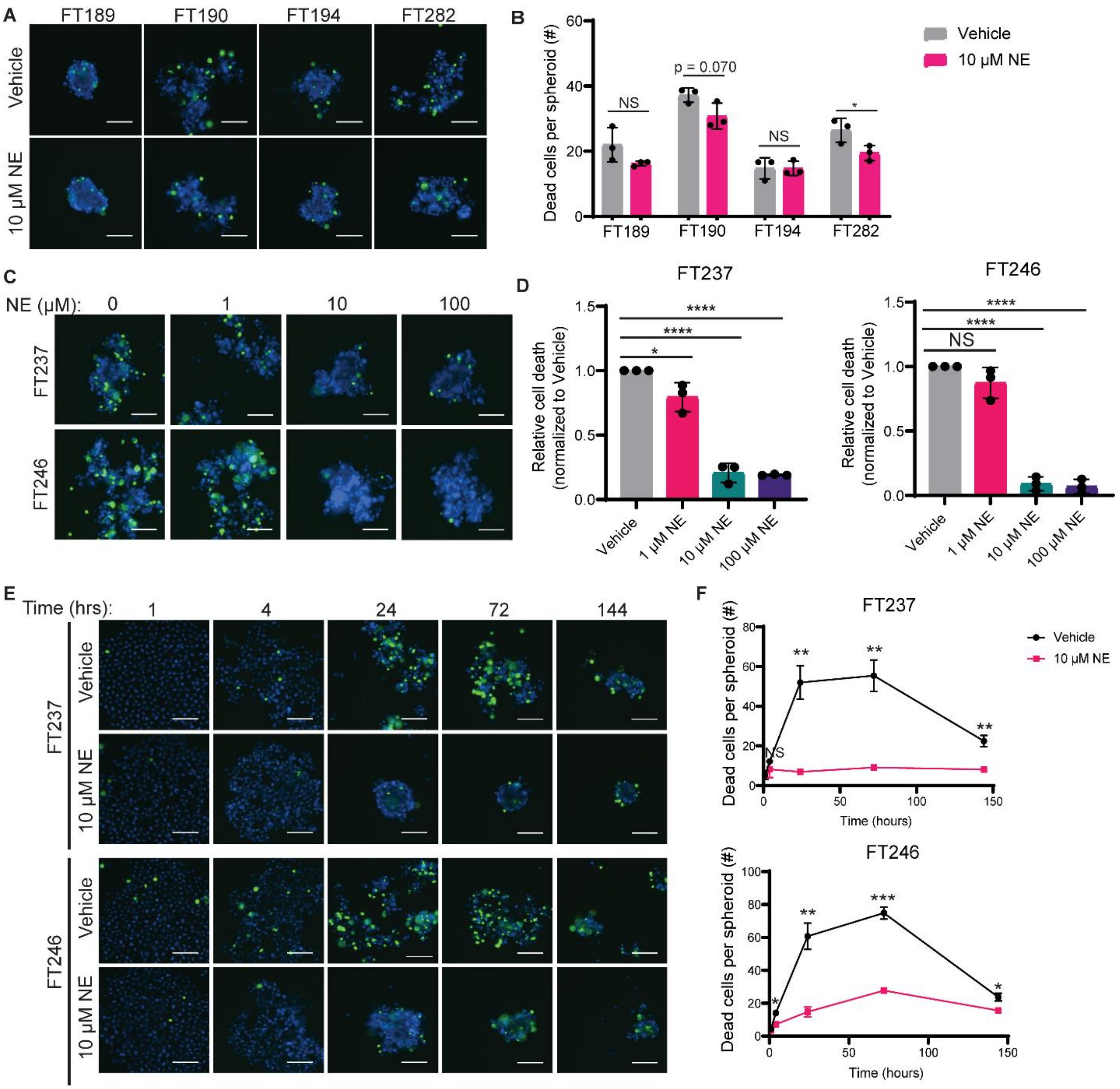

Supplemental Figure 2: Fluorescent images and quantification of dead cells (green) in spheroids (blue) cultured under ULA conditions. (**A,B**) 4 additional immortalized FTE cell lines (FT189, FT190, FT240, FT282) grown for 24 hrs -/+ 10μM NE, (**C,D**) FT237 and FT246 cells grown for 24 hrs in various concentrations of NE, (**E,F**) FT237 and FT246 cells grown -/+ 10μM NE at various timepoints. Statistical analyses were conducted via: (**B,F**) Student's t-tests, or (**D**) one-way ANOVA with Dunnett's correction for multiple comparisons. Data are represented as mean ± standard deviation.

Supplemental Figure 3

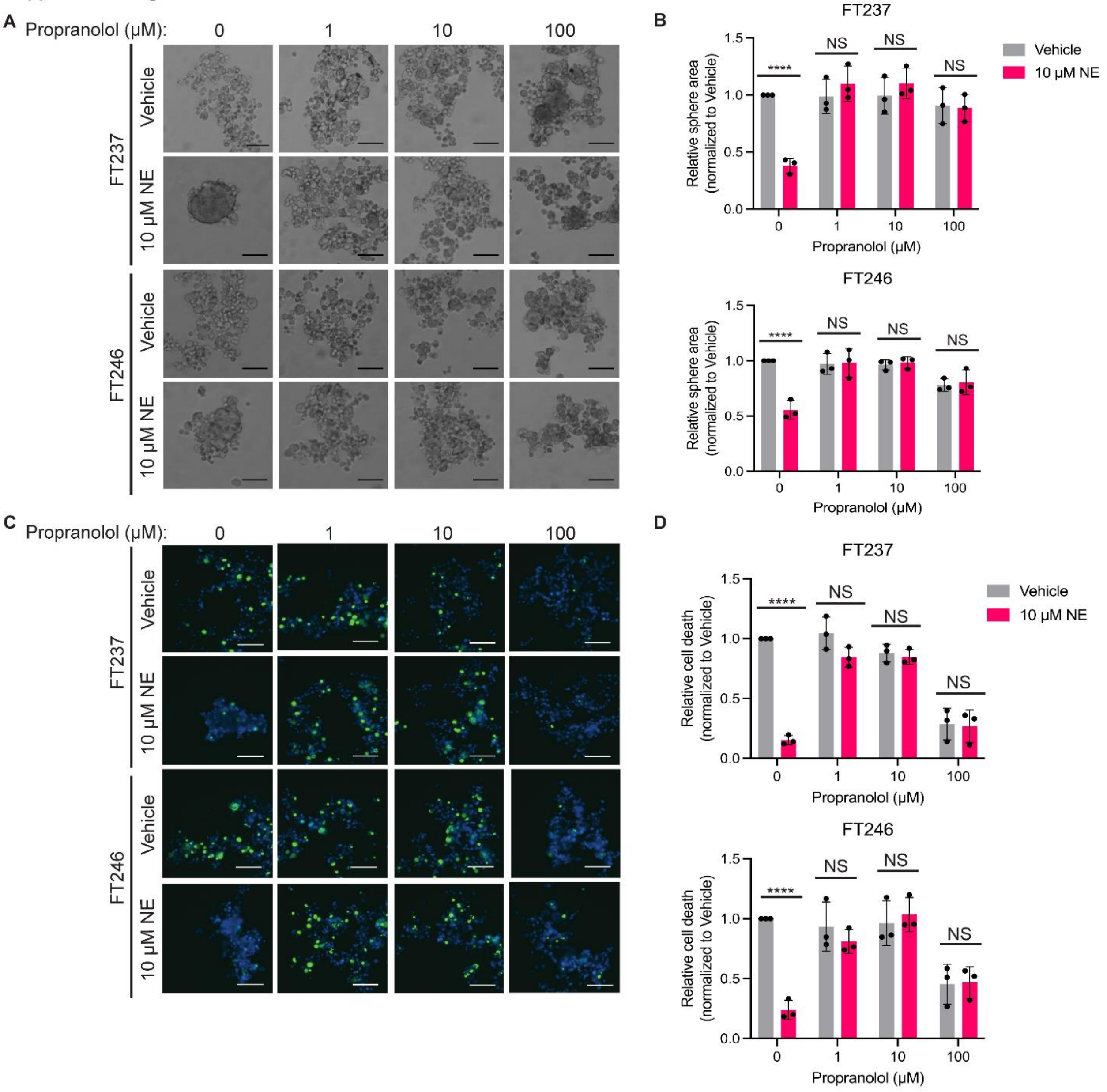

Supplemental Figure 3: (**A,B**) Brightfield images and quantification of spheroid areas for FT237 and FT246 cells cultured under ULA conditions for 24 hrs in various concentrations of propranolol. (**C,D**) Fluorescent images and quantification of dead cells (green) in spheroids (blue) as cultured in (**A**). All statistical analyses for this figure were conducted via two-way ANOVA with Sidak's correction for multiple comparisons. Data are represented as mean ± standard deviation.

Supplemental Figure 4

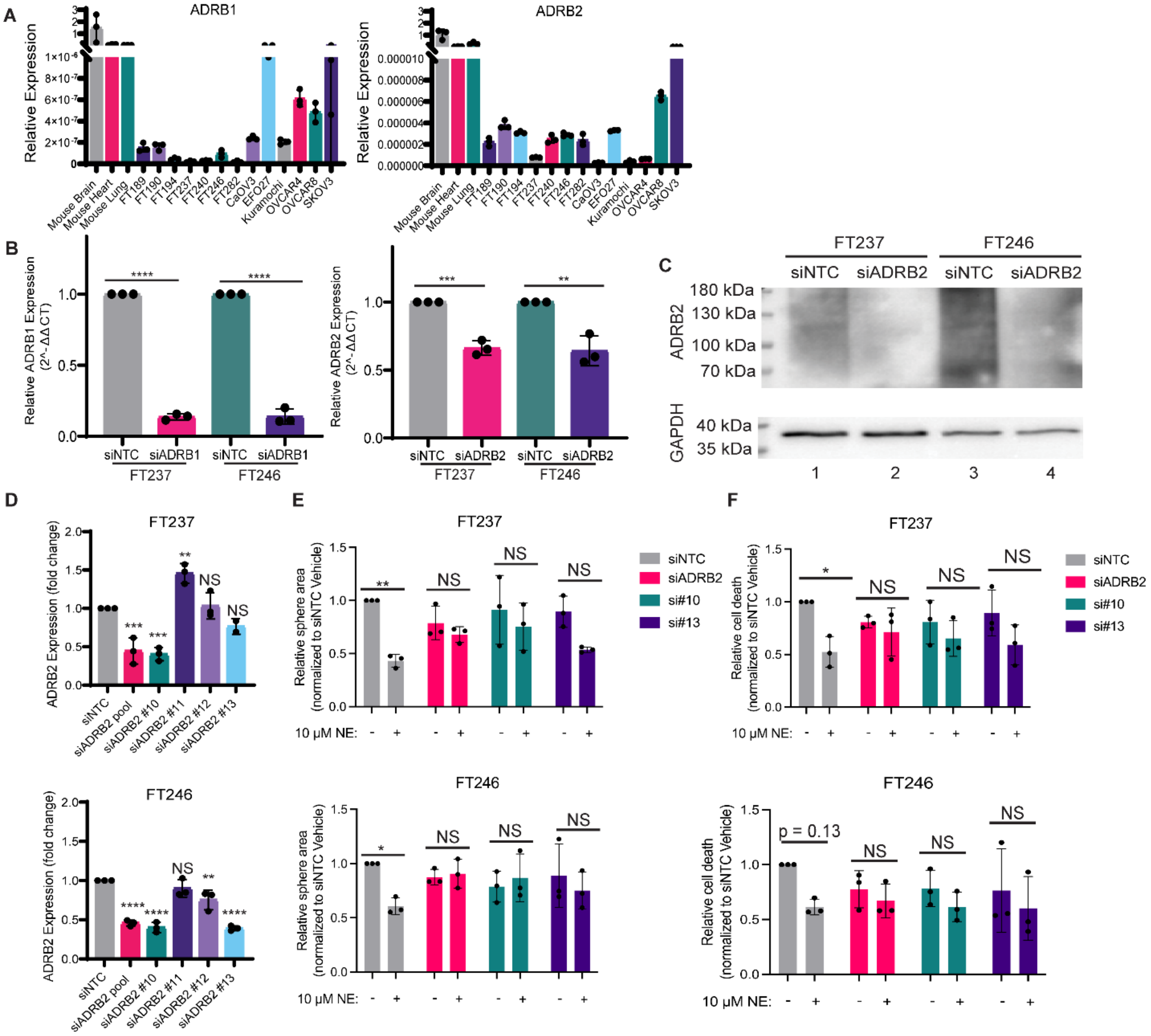

Supplemental Figure 4: (**A**) Relative RNA expression levels of ADRB1 and ADRB2 in control tissues and immortalized FTE and HGSC cell lines. (**B**) RTqPCR validation of knockdown for pooled siRNAs targeting ADRB1 and ADRB2 in FT237 and FT246. (**C**) Western blot validation of ADRB2 knockdown. (**D**) RTqPCR deconvolution of individual siRNAs within the siADRB2 pool in FT237 and FT246. (**E**) Quantification of relative changes in sphere areas upon knockdown of ADRB2 with pooled or individual siRNAs. (**F**) Quantification of relative changes in cell death for spheres described in (**E**). Statistical analyses were conducted via: (**B**) Student's t-tests, (**D**) one-way ANOVA with Dunnett's correction for multiple comparisons, and (**E,F**) two-way ANOVA with Sidak's correction for multiple comparisons. Data are represented as mean ± standard deviation.

Supplemental Figure 5

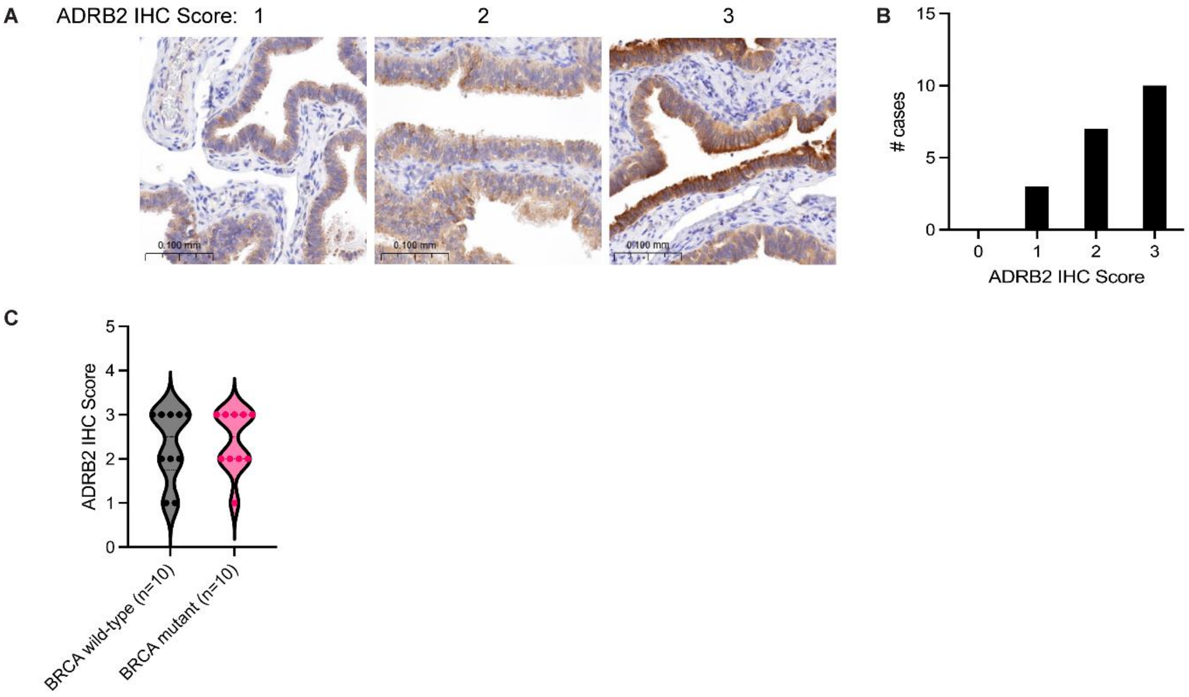

Supplemental Figure 5: (**A**) Representative images of H&E stained human fallopian tube epithelial tissue with low (1), medium (2) and high (3) ADRB2 staining (brown). (**B**) Distribution of ADRB2 staining intensity for the immunohistochemistry of samples in (**A**) (n=20). (**C**) Violin plots depicting ADRB2 staining levels according to BRCA status.

Supplemental Figure 6

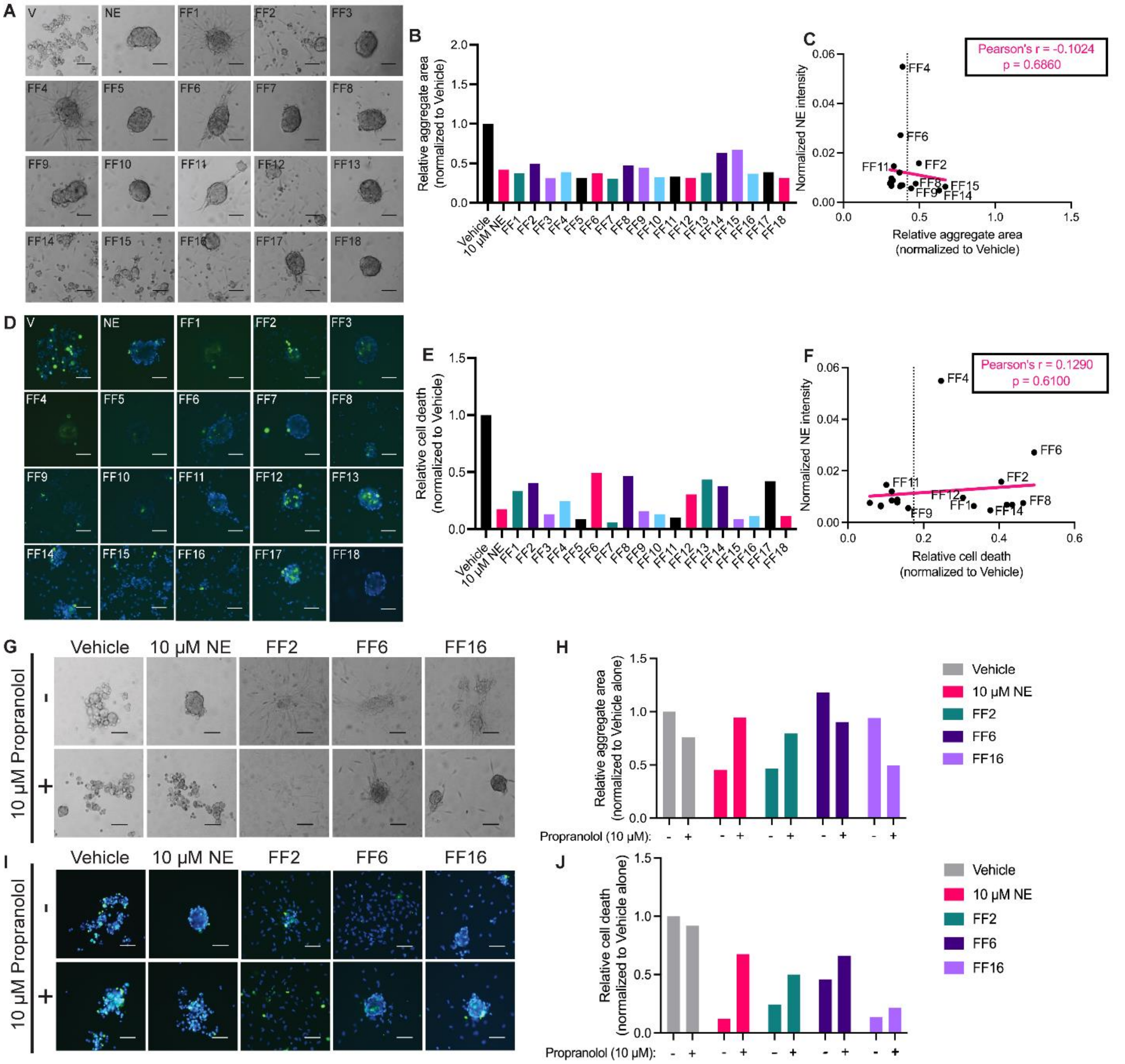

Supplemental Figure 6: (**A,B**) Brightfield images and area measurements for FT237 cells treated for 24 hrs in ULA with 18 different follicular fluid samples collected from 17 different patients (10% volume). (**C**) Linear regression for correlation between FT237 aggregate area and relative NE concentration quantified via mass spectrometry analysis. (**D,E**) Fluorescent images of total cells (blue) and dead cells (green) in each FT237 cell aggregate, cultured as in A. (**F**) Linear regression for correlation between the number of dead cells in FT237 aggregates and relative NE concentration per sample. (**G,H**) Brightfield images and area measurements for FT237 cells treated with vehicle, NE, FF2, FF6, or FF16 in the presence or absence of 10µM propranolol. (**I,J**) Fluorescent images and dead cell quantification for cells as cultured in (**G**). Each experiment was conducted in technical triplicate.

Supplemental Figure 7

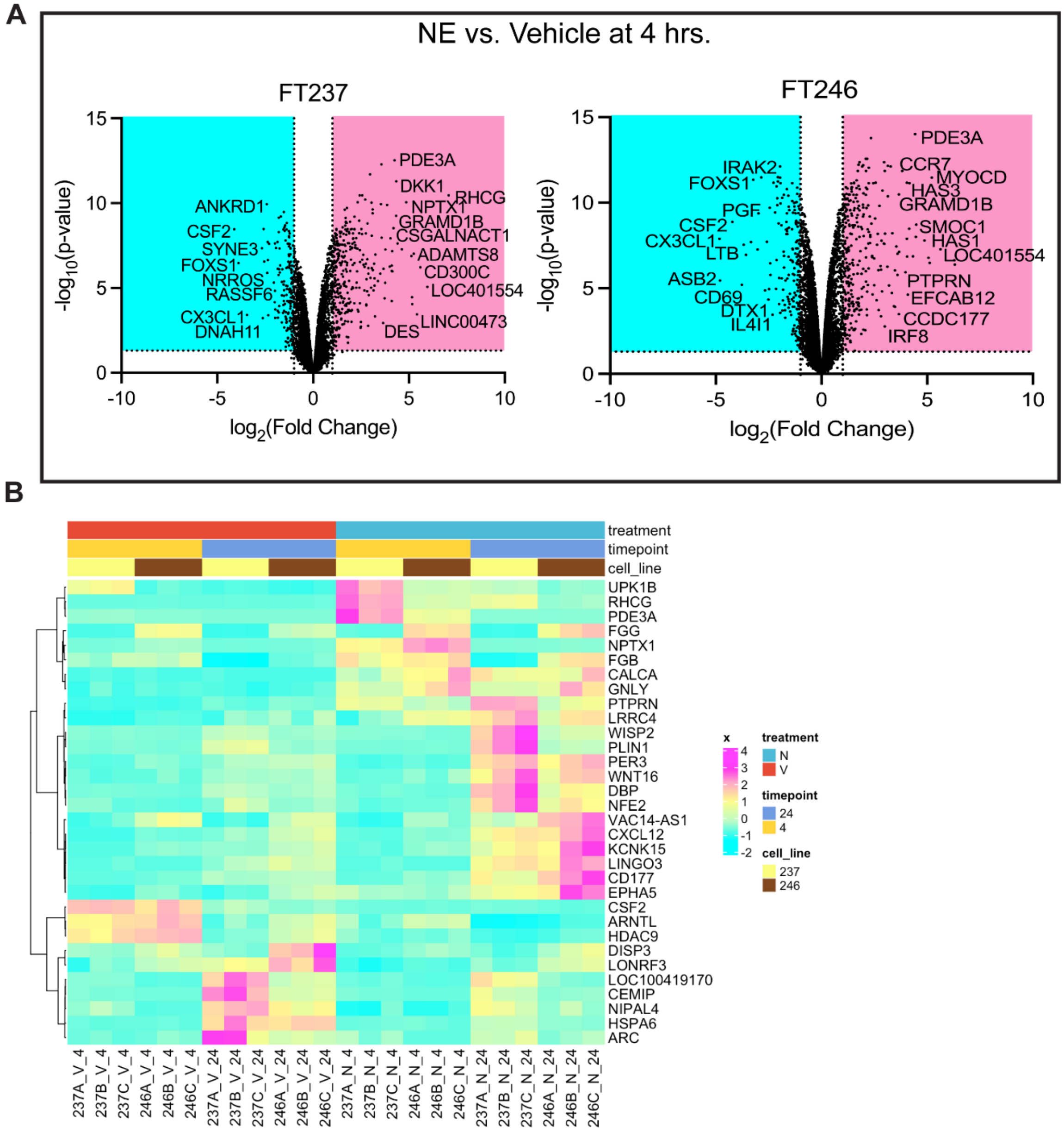

Supplemental Figure 7: (**A**) Volcano plots for differentially expressed transcripts in ULA-cultured FT237 and FT246 that are upregulated (magenta) and downregulated (cyan) after 4 hrs of 10μM NE treatment relative to vehicle. (**B**) Temporal heatmap for top differentially expressed transcripts common to both cell lines at early (4hr) and late (24hr) timepoints, relative to the baseline cell profiles prior to ULA culture.

Supplemental Figure 8

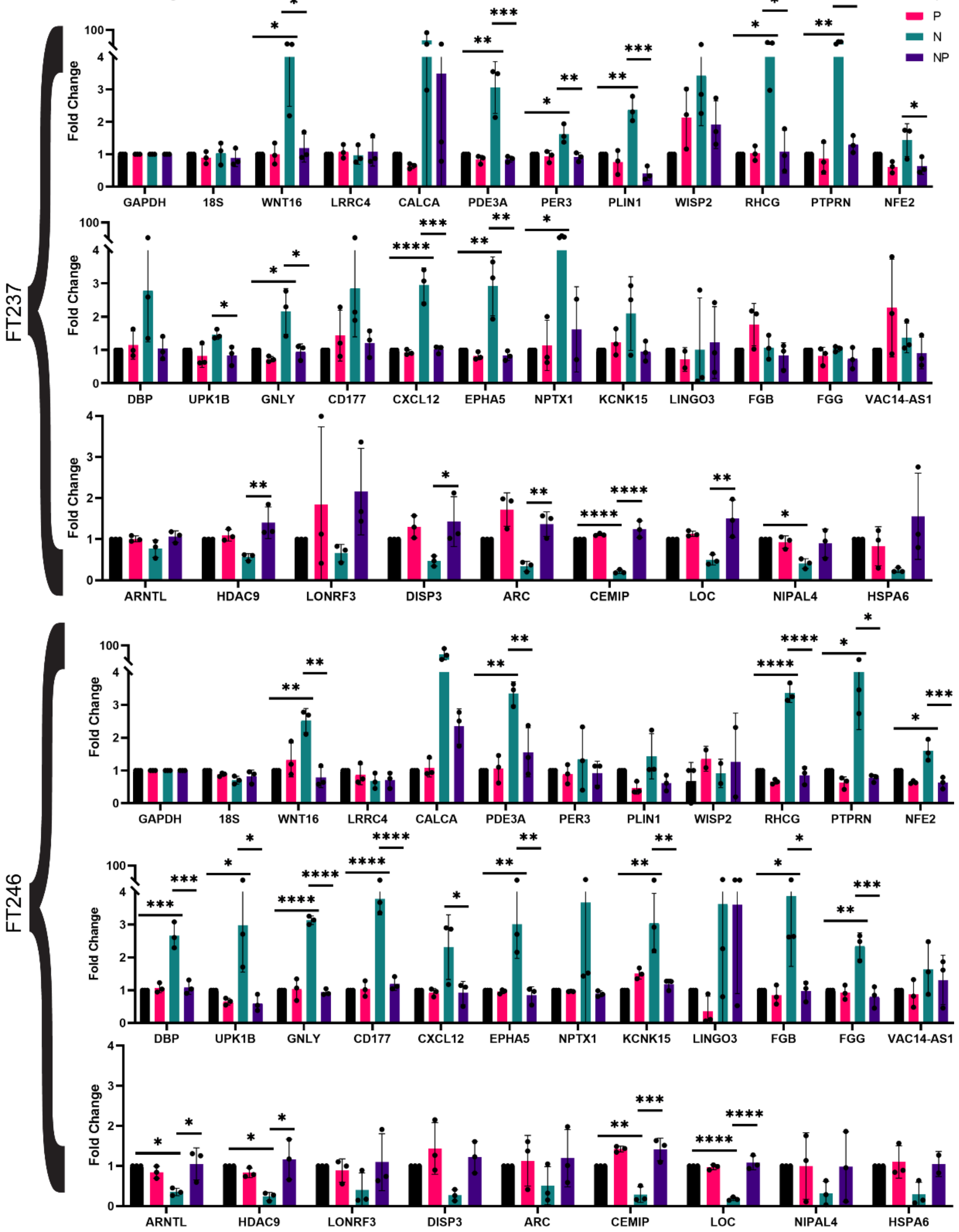

Supplemental Figure 8: RTqPCR validation of RNAsequencing hits common to both FT237 and FT246. All statistical analyses for this figure were conducted using one-way ANOVA with Tukey's correction for multiple comparisons. Data are represented as mean ± standard deviation.

Supplemental Figure 9

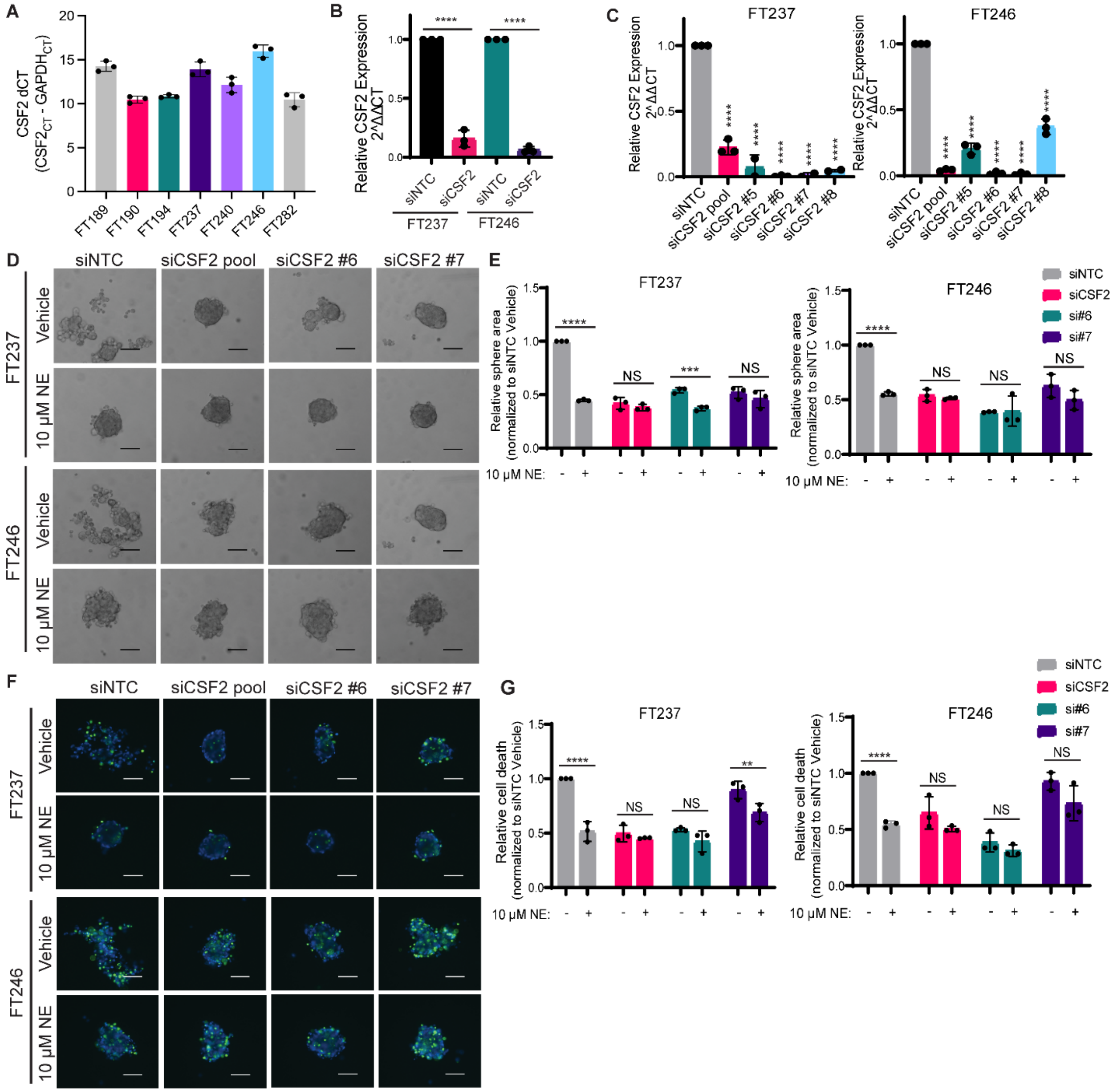

Supplemental Figure 9: (**A**) RTqPCR panel of CSF2 mRNA expression across FTE cell lines. (**B**) RTqPCR validation of knockdown for pooled siRNA targeting *CSF2* in FT237 and FT246. (**C**) RTqPCR deconvolution of individual siRNAs within the siCSF2 pool in FT237 and FT246. (**D,E**) Representative brightfield images and quantification of sphere areas for FTE cell lines transfected with two of the most efficient siRNAs (#6 and #7) when cultured under ULA conditions for 24 hrs in the presence or absence of 10μM NE. (**F,G**) Representative fluorescent images of total cells (blue) and dead cells (green) and quantification of relative cell death in each condition as cultured in (**D**). Statistical analyses were conducted via: (**B**) Student's t-tests, (**C**) one-way ANOVA with Dunnett's correction for multiple comparisons, or (**E, G**) two-way ANOVA with Sidak's correction for multiple comparisons. Data are represented as mean ± standard deviation.

Supplemental Table 1. List of primers used for RTqPCR experiments

Primer	Sequence (5' ---> 3')	Primer	Sequence (5' ---> 3')
GAPDH_F	GAGTCAACGGATTTGGTCGT	***PER3_F***	GCAGAGGAAATTGGCGGACA
GAPDH_R	TTGATTTTGGAGGGATCTCG	***PER3_R***	GGTTTATTGCGTCTCTCCGAG
ADRB1_F	CGAGACCCTGTGTGTCATTG	***CXCL12_F***	ATTCTCAACACTCCAAACTGTGC
ADRB1_R	ACGACTTGGGGTCGTTGTAG	***CXCL12_R***	ACTTTAGCTTCGGGTCAATGC
ADRB2_F	GAGCACAAAGCCCTCAAGAC	***LRRC4_F***	CTCCCGTTCGTCTACCTCAC
ADRB2_R	TGGAAGGCAATCCTGAAATC	***LRRC4_R***	GGTGTTCGAGGGAATACCCTG
18S_F	GGAAAGCAGACATTGACCTCAC	***FGG_F***	TTATTGTCCAACTACCTGTGGC
18S_R	CCATCCTTTACATCCTTCTGTCTGT	***FGG_R***	GACTTCAAAGTAGCAGCGTCTAT
CALCA_F	TGCAGATGAAGGCCAGTGAG	***FGB_F***	AGTGATTCAGAACCGTCAAGAC
CALCA_R	TACTCAGATTACCGCACCGC	***FGB_R***	CATCCTGGTAAGCTGGCTAATTT
RHCG_F	GGTTCCACTTCTTACAAGACCG	***LINGO3_F***	TGCCTCAATCTGTCGCACAA
RHCG_R	GGGCTGACTTTACCCAGAACT	***LINGO3_R***	CAGCGTCTCTAGCGTGTTCA
UPK1B_F	CCAAAGACAACTCAACTGTTCGT	***PLIN1_F***	CCATGTCCCTATCAGATGCCC
UPK1B_R	AATGCCGCAACAACCAATAATC	***PLIN1_R***	CTGGTGGGTTGTCGATGTC
GNLY_F	CCTGTCTGACGATAGTCCAAAAA	***VAC14AS1_F***	TACGAGGGTGTCCCAAAAGC
GNLY_R	GACCTCCCCGTCCTACACA	***VAC14AS1_R***	TTCCTGGATGCGGGAGACTA
CD177_F	ATGAGCGCGGTATTACTGCTG	***HSPA6_F***	CGTGCCCGCCTATTTCAATG
CD177_R	GGTCGGACACCTTCCACAC	***HSPA6_R***	AAAAATGAGCACGTTGCGCT
PDE3A_F	CCACGGCCTCATTACCGAC	***CSF2_F***	TCCTGAACCTGAGTAGAGACAC
PDE3A_R	TTGCTCACGGCTCTCAAGG	***CSF2_R***	TGCTGCTTGTAGTGGCTGG
NFE2_F	GCAGGAACAGGGTGATACAGC	***CEMIP_F***	GCTCTTGAGTTGCATGGACA
NFE2_R	GCAGCTCGGTGATGGACAT	***CEMIP_R***	ACCGCGTTCAAATACTGGAC
WNT16_F	TTCAGACACGAGAGATGGAACT	***LOC100419170_F***	GGAAGCCTGAAGCTCCCTAT
WNT16_R	CCAGCCTTCACTTGCTGAG	***LOC100419170_R***	CCTCGTGTCTGGGAAACAAT
KCNK15_F	GCTCTCCGGAGGAAGTTCG	***DISP3_F***	CACAGCCTGCAGAACAACAT
KCNK15_R	TGATGACGGTGATGGCGAAG	***DISP3_R***	AGGCGCTCTATACACCTGGA
PTPRN_F	CGGGACACATGATTCTGGCAT	***HDAC9_F***	AGTAGAGAGGCATCGCAGAGA
PTPRN_R	CTGCTTGGTAGGCACAGAGG	***HDAC9_R***	GGAGTGTCTTTCGTTGCTGAT
NPTX1_F	CACCGAGGAGAGGGTCAAGAT	***ARNTL_F***	AAGGGAAGCTCACAGTCAGAT
NPTX1_R	CAGGGCGGTTGTCTTTCTGA	***ARNTL_R***	GGACATTGCGTTGCATGTTGG
WISP2_F	CCTCCTCTCAAAGGTGCGTA	***NIPAL4_F***	CCAACTTTGGAGCCTACGCAT
WISP2_R	CTGTCGTCCTCTGCCAAGA	***NIPAL4_R***	TCCATGATGGTAGTGACCTTCT
DBP_F	CTGATCTTGCCCTATCAAGCATT	***LONRF3_F***	ATGGAGTCAGTACGGATCGAA
DBP_R	CGATGTCTTCGAGGGTCAAAG	***LONRF3_R***	GTCCCCGGAGACTGCTCTT
EPHA5_F	TAGGACCTCTAAGCAAAAAGGGA	***ARC_F***	AGCGGGACCTGTACCAGAC
EPHA5_R	GAGCCTGACACTTCGAGCAA	***ARC_R***	GCAGGAAACGCTTGAGCTTG
