# Supplementary figures and images for: Norepinephrine induces anoikis resistance in high-grade serous ovarian cancer precursor cells
Source: JCI Insight. 2024 Jan 25;9(5):e170961. doi: 10.1172/jci.insight.170961 (PMC10972597; doi:10.1172/jci.insight.170961)

**Full unedited blots for Supplemental Figure 4:**

**ADRB2**  
Sigma #HPA003431  
(1:1000)

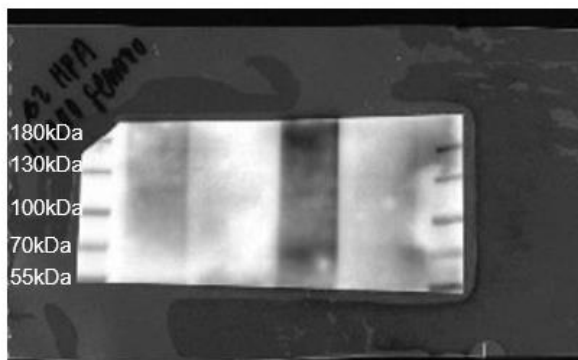

**GAPDH**  
CST #97166S  
(1:1000)

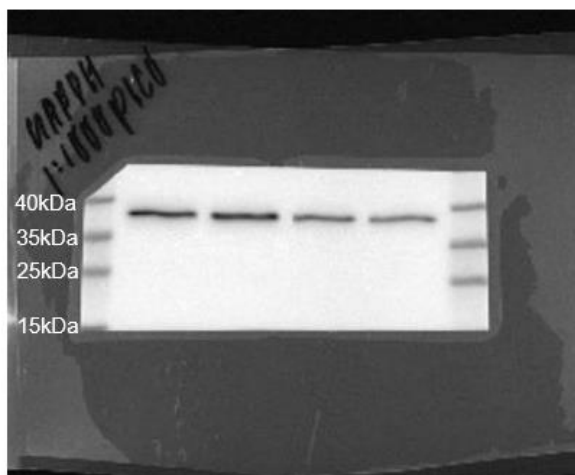

Supplement: Unedited blot and gel images [file jciinsight-9-170961-s044.pdf]
